# Engineering CAR‐Macrophages With Advanced Delivery Systems for Tissue Repair

**DOI:** 10.1002/advs.77234

**Published:** 2026-08-19

**Authors:** Yixin Zhang, Jintian Yu, Liang Yuan, Ziliang Fu, Yiming Yao, Lingna Chen, Shixiong Chen, Aikemu Ainiwaer, Xiaolin Cui

**Affiliations:** ^1^ Department of Biomedical Engineering School of Medicine The Chinese University of Hong Kong Shenzhen China; ^2^ School of Life Sciences Xinjiang Normal University Urumqi China

**Keywords:** CAR‐Macrophages, delivery system, lipid nanoparticles, tissue engineering and regenerative medicine

## Abstract

Tissue injury and organ dysfunction remain major clinical challenges, as conventional therapies often fail to achieve functional regeneration. Chimeric antigen receptor (CAR) technology endows macrophages with the ability to specifically recognize and clear pathological targets, making CAR‐macrophages (CAR‐M) a promising tool in tissue engineering and regenerative medicine. However, the efficient, safe, and controllable engineering of CAR‐M still depends on advanced chemical delivery systems. This review systematically summarizes five major platforms for CAR‐M engineering, including viral vectors, lipid nanoparticles (LNPs), exosomes/extracellular vesicles, polymeric nanocarriers, and biomaterial scaffolds. Particular emphasis is placed on LNPs optimization strategies, including ionizable lipid design, surface modification, and regulation of physicochemical properties. The influence of delivery systems on macrophage uptake, intracellular trafficking, and polarization is also discussed. This review further highlights recent preclinical applications of CAR‐M therapy in liver fibrosis, cardiac fibrosis, and atherosclerosis. Furthermore, a comparative analysis of CAR‐M with CAR‑T and CAR‑NK therapies is provided, and key challenges, including phenotypic instability, off‑target effects, and limited in vivo persistence, are discussed. Finally, future directions are outlined, including advanced delivery strategies, multi‑target CAR designs, and metabolic modulation, highlighting new opportunities for precision regenerative immunotherapy.

## Graphical abstract

This review highlights how engineered macrophages equipped with chimeric antigen receptors (CAR) guide tissue repair by recognizing disease‐related targets, clearing harmful cells, and reshaping local immune environments. It summarizes macrophage biology, CAR design, delivery platforms, and functionalization strategies, and discusses emerging applications in liver fibrosis, cardiac fibrosis, and atherosclerosis, alongside translational challenges for regenerative medicine. OXPHOS: oxidative phosphorylation; ECM: extracellular matrix

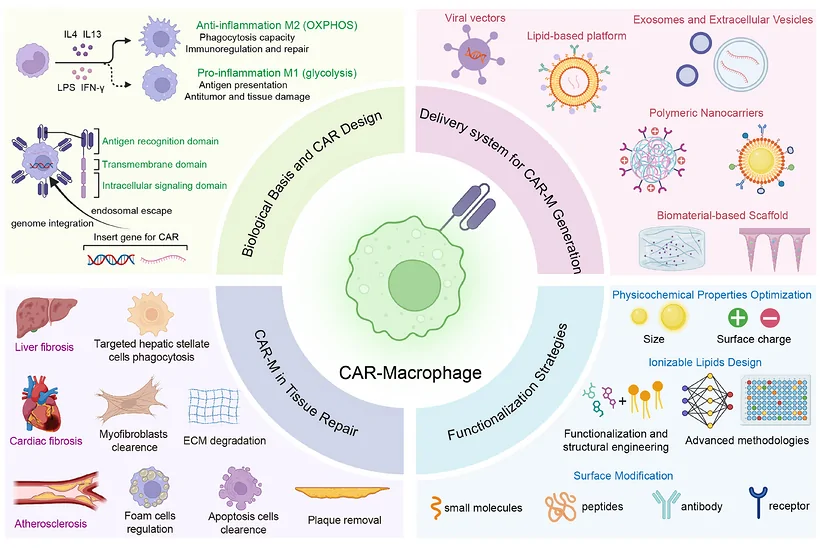

## 1 Introduction

Tissue and organ injury, together with the resulting functional loss caused by trauma, ischemic diseases, degenerative lesions, and congenital malformations, represents a major global health burden [1]. This challenge is particularly pronounced in high‐mortality non‐communicable diseases, including cardiovascular and fibrotic disorders [1]. Conventional interventions, such as surgical repair, artificial prosthesis implantation, and organ transplantation, are often limited by inadequate long‐term functional integration, immune rejection, and donor shortage, making them insufficient to meet growing clinical demand [2, 3]. Tissue engineering and regenerative medicine have therefore emerged, with the primary aim of repairing, replacing, or enhancing damaged tissues and organ function through the interdisciplinary integration of biology, engineering, and materials science [4]. Although substantial progress has been made in stem cell transplantation, biomaterial scaffold construction, and growth factor delivery, clinical translation continues to face a fundamental challenge: the dynamic interactions between implants and the host immune system [5, 6]. Traditionally, immune responses have been regarded primarily as a barrier to transplant success and therefore need to be suppressed through immunosuppressive strategies [7]. However, this view has gradually shifted. Increasing evidence indicates that innate immune cells play indispensable regulatory roles in tissue repair, angiogenesis, and organ remodeling [8, 9]. Hence, establishing a properly coordinated immune microenvironment is now recognized as essential for achieving functional regeneration rather than fibrotic scar formation [10].

Among innate immune cells, macrophages are particularly important because of their remarkable plasticity and functional heterogeneity [11]. They contribute to tissue homeostasis, pathogen clearance, injury repair, and tumor immune surveillance. In addition to serving as highly efficient phagocytes that eliminate debris, pathogens, and apoptotic cells, macrophages actively regulate inflammation, angiogenesis, cell proliferation, and extracellular matrix (ECM) remodeling through secretion of cytokines, chemokines, growth factors, and proteases [12]. Owing to these diverse functions, macrophages have emerged as highly compelling targets and cellular vehicles for immune‐engineering applications in tissue engineering and regenerative medicine [13]. However, conventional approaches for macrophage modulation, such as nanoparticle‐based or soluble cytokine delivery, often lack sufficient spatiotemporal precision and cell specificity, limiting their ability to precisely control the local immune microenvironment in complex in vivo settings.

The emergence of Chimeric antigen receptor (CAR) technology provides a revolutionary tool to address this issue [14, 15]. CAR engineering integrates a specificity‐determining antigen‐recognition domain, typically a single‐chain variable fragment (scFv), with intracellular signaling domains, therefore endowing host cells with non‐MHC‐restricted targeting and activation capabilities [16]. Although CAR‐T cell therapy has achieved tremendous success in hematologic malignancies, its application in non‐malignant contexts is limited by the difficulty of T cells penetrating dense tissues and by their lack of intrinsic phagocytic and tissue‐remodeling functions [17, 18]. In contrast, macrophages naturally possess tissue tropism, phagocytic activity, antigen‐presenting capacity, and the ability to remodel local microenvironments across multiple tissues [19]. These properties give CAR‐macrophages (CAR‐M) unique advantages for tissue repair and regeneration. Nevertheless, major challenges remain, particularly in how to deliver CAR elements to macrophages efficiently and safely, and how to regulate their behavior precisely within specific tissue microenvironment.

To address these issues, increasing attention has been directed toward the development of advanced delivery systems, particularly lipid nanoparticles (LNPs), polymeric nanocarriers, and natural vesicles. These platforms provide versatile and controllable vehicles for CAR‐M generation, either through ex vivo engineering followed by adoptive transfer or, more attractively, through in vivo in situ reprogramming of endogenous macrophages [20, 21]. Owing to their tunable physicochemical properties, surface modification, biocompatibility, and controllable release, these nanocarriers can not only protect genetic material such as mRNA and plasmids to enable CAR expression, but also co‑deliver additional therapeutic cargos, including small‑molecule drugs, siRNA, and growth factors, to synergistically modulate macrophage phenotype and function [22].

**FIGURE 1:**
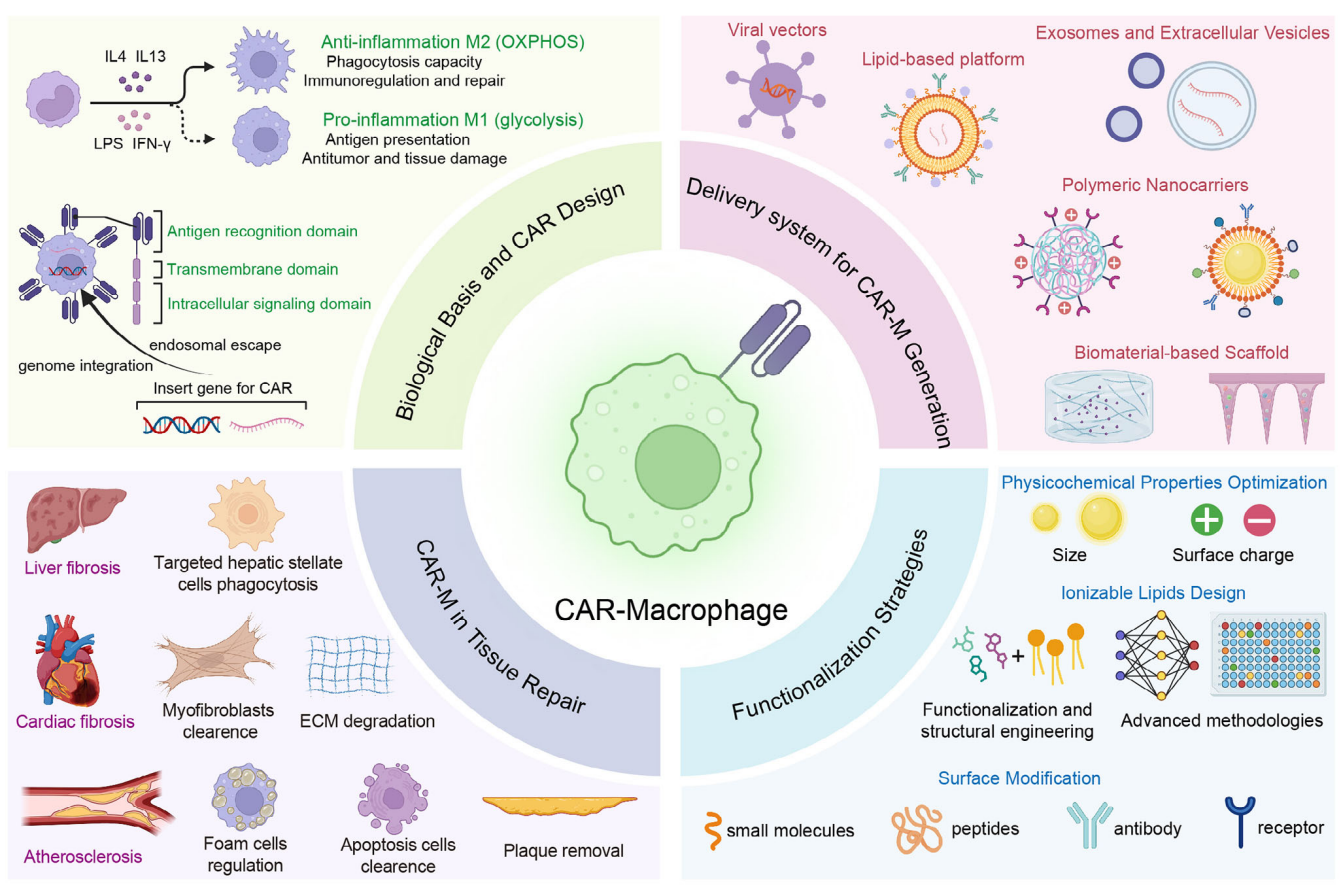
This schematic illustrates the biological basis and design principles of CAR‐M, including macrophage polarization states and their functions. It also summarizes five promising delivery systems for CAR elements transfer, along with optimization strategies. Furthermore, the schematic presents direct applications of CAR‐M in tissue repair, involving liver fibrosis, cardiac fibrosis, and atherosclerosis. Created in BioRender. Cui, S. (2026) https://BioRender.com/co780cc.

Despite the rapidly growing interest in CAR‐M, most existing reviews have focused primarily on oncology, whereas comprehensive discussion of nanocarrier design and CAR‐M applications in tissue repair remains limited. Herein, this review aims to systematically illustrate the design principles, therapeutic applications, and current challenges of CAR‑M in tissue engineering and regenerative medicine, with particular emphasis on the pivotal role of nanocarrier‐based delivery systems (Figure 1). We first summarize macrophage biology and the action mechanisms of CAR‑M. We then discuss the major delivery systems used for CAR‐M engineering, with a focus on optimization approaches and functionalization strategies. Because the evidence supporting CAR‐M‐mediated tissue repair remains limited across disease contexts, we also consider related studies involving general macrophage delivery, CAR‐M immunotherapy, and non‐CAR macrophage engineering, as these may inform future tissue‐repair strategies. In addition, we highlight recent advances in CAR‐M applications for liver and cardiac repair. Finally, we discuss key safety concerns, translation challenges, and future directions for the development of next‑generation precision regenerative immunotherapies. Overall, this review provides a timely framework for advancing the novel design and translational development of CAR‐M‐based therapies for tissue repair and regeneration.

## 2 Biological Basis and Mechanisms of CAR‐M

Macrophages are a heterogeneous population derived from myeloid progenitor cells, which exhibit remarkable plasticity in their functions within tissue microenvironments [23]. Traditionally, they have been classified into M1 and M2 subtypes based on their activation state [24]. M1 macrophages, typically induced by lipopolysaccharide (LPS) and interferon‐γ (IFN‐γ), display pro‐inflammatory, bactericidal, and anti‐tumor properties [23]. They clear pathogens and damaged tissues passively by producing inflammatory cytokines such as tumor necrosis factor‐alpha (TNF‐α), interleukin‐1 beta (IL‐1β), and interleukin‐6 (IL‐6), along with nitric oxide (NO) and reactive oxygen species (ROS) [25]. Conversely, M2 macrophages, induced by interleukin‐4 (IL‐4) and interleukin‐13 (IL‐13), are involved in inflammation resolution, immunomodulation, tissue repair, and remodeling [26]. Their characteristics include high expression of anti‐inflammatory cytokines like interleukin‐10 (IL‐10) and transforming growth factor‐β (TGF‐β), as well as repair‐associated genes such as arginase‐1 (Arg1) and CD206 [27]. However, this simplistic M1/M2 classification has gradually been superseded by more nuanced models of continuous polarization spectra, acknowledging that macrophages can adopt diverse hybrid phenotypes under different stimuli and that distinct functional subsets may coexist within the same tissue [19].

During the tissue repair process, the dynamic plasticity of macrophages is indispensable. Early post‐injury, M1‐like macrophages clear apoptotic cells and pathogens, initiating an inflammatory response [28]. Subsequently, macrophages shift toward an M2‐like phenotype, promoting inflammation resolution, stimulating fibroblast proliferation, collagen deposition, and angiogenesis, ultimately facilitating tissue remodeling [29]. For instance, during fracture and osteoarthritis healing, macrophages contribute to hematoma clearance, endochondral ossification, and bone repair [30, 31]. In skin wounds, they modulate collagen synthesis, angiogenesis, and epidermal regeneration [32]. Therefore, precisely regulating macrophage polarization states and functions is significant for optimizing tissue regeneration.

However, achieving such precise regulation in vivo remains a huge challenge using traditional pharmacological or soluble cytokine‐based approaches, which often suffer from rapid clearance and off‐target effects. To surmount this bottleneck, synthetic biology, specifically CAR technology, has been leveraged to engineer macrophages with user‐defined specificities and controllable effector functions [33].

CARs are synthetic membrane receptors that confer exogenous antigen‐recognition capabilities to macrophages and dictate the intracellular signaling networks and effector responses triggered upon target antigen recognition [34]. A typical CAR structure comprises three components:

(1) Extracellular Binding Domain: Usually a scFv, this domain specifically recognizes particular antigens on the surface of target cells or within the ECM. This domain is responsible for guiding macrophages to the target site and initiating the recognition process [35].

(2) Transmembrane Domain: This domain links the extracellular binding domain to the intracellular signaling domain and anchors the CAR molecule to the cell membrane. Its sequence selection influences CAR's stable expression and signal transduction efficiency [36].

(3) Intracellular Signaling Domain: This domain transmits antigen recognition signals into the cell, activating macrophage effector functions [33]. It is the critical component in CAR‐M design, as it determines the specific biological responses of activated macrophages. Early CAR‐M designs often adapted signaling domains from CAR‐T cells, such as the CD3ζ chain, but studies suggest CD3ζ alone may be insufficient for robust phagocytic activation in macrophages [33]. Consequently, novel CAR‐M signaling domain designs increasingly incorporate macrophage‐specific activation pathways to effectively enhance their capabilities. For example, CAR‐M engineered with CD40 or TLR4 signaling domains demonstrate enhanced pro‐inflammatory phenotype maintenance, conferring an advantage in anti‐tumor therapy [37]. Conversely, by combining specific signaling domains like MerTK, CAR‐M can be facilitated to start the gentle clearance of apoptotic cells via efferocytosis, which is conducive to tissue repair [38].

When CAR‐M bind to the target antigen via the extracellular recognition domain, the intracellular signaling domain is activated, initiating downstream signaling cascades. This activation not only enhances the phagocytic activity of macrophages to clear apoptotic or aberrant cells, but can also induce the release of cytokines, modulate chemokine secretion, and even alter their own metabolic state and gene expression profile [39]. In the field of tissue engineering and regenerative medicine, this programmable activation drives therapeutic outcome through two synergistic mechanisms:

(1) Enhance phagocytosis to directly mediate tissue repair: CAR‐M can improve the recognition of specific pathological targets, thereby clearing necrotic tissue and cellular debris at injury sites. This creates a more favorable local environment for the subsequent repair process. In specific disease contexts, this function can be extended to the targeted elimination of abnormal cells or structures. For example, it can identify and phagocytose pro‐fibrotic fibroblasts or target and clear pathological amyloid‐β, directly intervening in disease progression [40, 41].

(2) Modulate the local immune microenvironment and promote regeneration: Beyond direct clearance, M2‐type CAR‐M can reshape the microenvironment of injured tissues by secreting various cytokines, growth factors, and immunomodulatory molecules [42]. Collectively, these functions contribute to local inflammation resolution, coordinated intercellular signaling, and ECM remodeling, thereby supporting tissue regeneration [43].

It should be noted that the reparative functions of CAR‐M are not exerted in a static or ideal environment, but are continuously shaped by the complex disease microenvironment. Injured tissues are commonly characterized by hypoxia, lactate accumulation, acidic pH, oxidative stress, and dysregulated lipid metabolism [44, 45, 46, 47]. These metabolic stresses can alter macrophage energy utilization and thereby influence their polarization, functional stability, and therapeutic efficacy [48]. In general, M1‐like pro‐inflammatory macrophages rely predominantly on glycolysis to rapidly support energy production and biosynthesis, whereas M2‐like pro‐reparative macrophages depend more on oxidative phosphorylation (OXPHOS) and fatty acid oxidation to sustain long‐term repair and tissue remodeling [49, 50].

Hypoxia is one of the most common metabolic stresses in injured tissues [51]. Persistent hypoxia may impair mitochondrial function, restrict cellular energy supply, and affect the migration, phagocytosis, and cytokine secretion of CAR‐M [52]. In fibrotic or chronic inflammatory diseases, these constraints may reduce the ability of CAR‐M to clear necrotic tissue, pathological ECM, and aberrantly activated fibroblasts, thereby weakening tissue remodeling and regenerative microenvironment regulation.

Lactate accumulation may further reshape CAR‐M behavior. Lactate acts not only as a metabolic byproduct but also as an immunoregulatory signal [53]. High lactate levels can suppress pro‐inflammatory responses and promote macrophage polarization toward M2 phenotype [54]. Although moderate reparative polarization may support inflammation resolution and regeneration, premature or excessive phenotypic transition may impair the targeted clearance capacity of CAR‐M. Therefore, the effect of lactate on CAR‐M therapy may be context‐dependent.

Dysregulated lipid metabolism represents another important metabolic barrier. In lipid‐rich pathological settings such as atherosclerosis and fatty liver disease, macrophages can take up large amounts of oxidized lipids and cholesterol, develop foam cell‐like phenotypes, and undergo endoplasmic reticulum stress [55, 56]. These changes may disrupt macrophage phagocytosis, cholesterol efflux, and inflammatory regulation [55]. For CAR‐M used in plaque remodeling or fibrotic intervention, such lipid burden may attenuate engineered functions and increase the risk of phenotypic drift.

Therefore, the therapeutic efficacy of CAR‐M depends not only on CAR architecture and target antigen recognition, but is also continuously regulated by the metabolic microenvironment. Based on this understanding, future CAR‐M design should incorporate metabolic adaptability as an important consideration. This may be achieved by selecting appropriate intracellular signaling domains to modulate macrophage metabolism and polarization, or by applying metabolic engineering approaches to improve mitochondrial fitness.

To this end, the rational design and generation of CAR‐M are fundamentally based on a deep understanding of macrophage biology. Further considerations dictate that these requirements, stemming from macrophage biology, need to be realized through appropriate delivery systems. Whether applied to antitumor therapy or tissue repair, this imposes distinct demands on vector type, delivery efficiency, expression kinetics, and biosafety. Crucially, the delivery vector is not merely a passive vehicle but actively participates in and shapes the functional state of the macrophage. Its chemical composition, surface properties, and interaction mechanisms with the cells can influence macrophage metabolism, polarization, and immune responses, thereby further determining the ultimate therapeutic performance of CAR‐M. Consequently, developing efficient and controlled delivery systems is the critical link between biological foundation and engineered applications.

## 3 Delivery System and Engineering Strategies

### 3.1 Delivery and Material Systems

The therapeutic applications of CAR‐M are continuously expanding, moving beyond traditional cancer immunotherapy to encompass broader disease areas such as tissue repair and regenerative medicine [57]. Whether through ex vivo genetic modification and reinfusion, or direct in vivo in situ generation of CAR‐M, the core lies in the rational selection and further optimization of the delivery system [58]. The significance of the delivery strategy extends beyond encapsulating and transporting CAR‐encoding elements. It is crucially important to ensure efficient entry into target macrophages and achieve effective expression, while minimizing immunogenicity and systemic toxicity.

**FIGURE 2:**
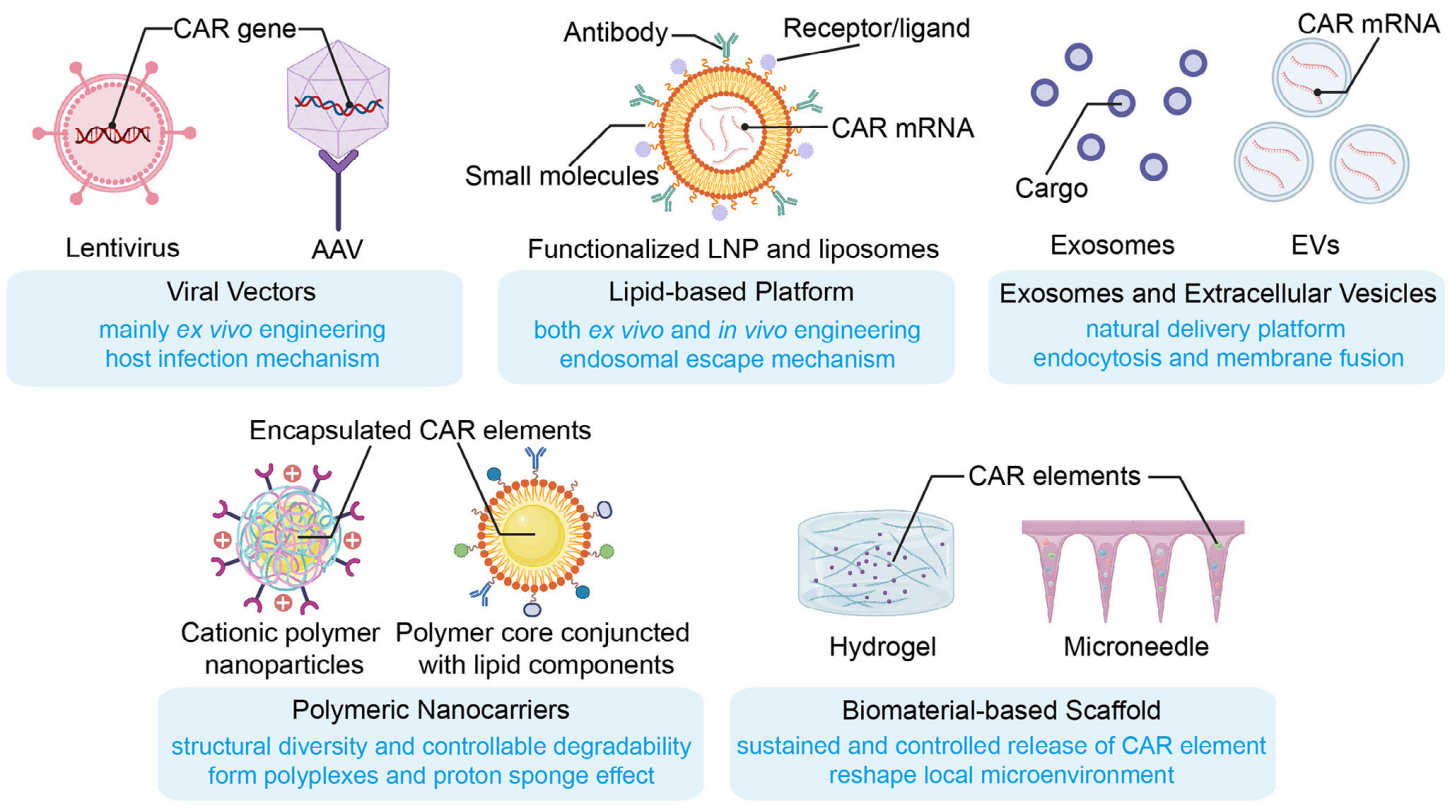
The five main delivery systems for CAR‐M engineering. Viral vectors include lentivirus and adeno‐associated virus (AAV) and are mainly used in ex vivo engineering. The lipid‐based platform includes LNPs and liposomes. LNPs are mainly used for in situ generation of CAR‐M and have multiple optimization strategies. Exosomes and extracellular vesicles are naturally derived delivery systems with low immunogenicity, excellent biocompatibility, and inherent targeting capability. Polymeric nanocarriers have structural diversity, controllable degradability, and ease of functionalization properties. They are often employed in conjunction with lipid components and may offer useful design flexibility for future CAR‐M delivery systems. Biomaterial‐based scaffold plays a multidimensional role and is the key part of tissue engineering. Created in BioRender. Cui, S. (2026) https://BioRender.com/co780cc.

Currently, delivery technologies built around CAR‐M have evolved along several key pathways, primarily encompassing viral vectors, lipid‐based platforms, natural nanocarriers (such as exosomes), synthetic polymer nanoparticles, and biomaterial scaffolds (Figure 2). Each type of delivery platform possesses distinct advantages and limitations regarding gene loading capacity, transduction or transfection efficiency, cellular targeting, biosafety, and application scenarios. They collectively form a diversified technical system that spans both ex vivo engineered modification and in vivo in situ programming. The rational selection and optimization of different delivery platforms based on specific therapeutic objectives, thereby enabling precise construction and functional regulation of CAR‐M, have emerged as critical steps in advancing this field toward clinical translation.

#### 3.1.1 Viral Vector

Viral vectors are among the earliest delivery systems used for CAR‐M and are particularly well suited to ex vivo engineering [59]. The first HER2‐targeted CAR‐M product to enter FDA clinical trials (CT‐0508) adopts this technology [60]. Although this particular construct was developed for solid tumor indications rather than tissue repair, its clinical safety profile nonetheless offers valuable reference for future regenerative applications. The core principle leverages the virus's intrinsic cell‐entry mechanisms to efficiently deliver exogenous CAR‐encoding elements into target cells. Specifically, adeno‐associated virus (AAV) vectors bind to cell‐surface receptors via capsid proteins and then be internalized in the receptor‐mediated endocytosis manner [61]. During endosomal acidification, conformational changes promote endosomal membrane rupture and the release of the viral genome into the cytoplasm, after which DNA is transported to the nucleus through the nuclear pore complex [62]. Because the genome typically does not integrate into the host chromosome, AAV vectors exhibit higher safety and are widely used in clinical trials worldwide [59]. However, the most significant constraint of AAV vector is the limited packaging capacity [63].

Lentiviral vectors principally rely on envelope glycoprotein–mediated fusion with the target cell membrane to release the viral genome directly into the cytoplasm [63]. The RNA genome is subsequently reverse‐transcribed into double‐stranded DNA and integrated into the host genome by integrase, enabling stable and long‐term CAR expression [64]. This makes lentiviral vectors more suitable for applications requiring prolonged CAR‐M persistence. For example, Liu et al. employed the lentiviral system to generate ex vivo CAR‑M capable of recognizing dipeptidyl peptidase 4 (DPP4)‑positive fibroblasts, thereby markedly attenuating scar formation after skin injury [65]. Similarly, Zhao et al. utilized the GV400 lentiviral vector to produce CAR‑M targeting FAP‑positive fibroblasts and secreting IL‑4 to maintain an M2 phenotype, resulting in mitigation of renal fibrosis [66]. However, this long‐term integration feature also carries risks of insertional mutagenesis and genomic safety concerns, necessitating careful assessment.

Overall, viral vectors have demonstrated feasibility and application potential in CAR‐M preclinical studies and early clinical exploration, yet they continue to face practical challenges, including complex manufacturing processes, stringent quality‐control requirements, and potential safety risks. Consequently, the development of safer, more versatile, and scalable non‐viral delivery systems has emerged as a key research direction in the CAR‐M field [67].

#### 3.1.2 Lipid‐Based Platform

Lipid‐based platform represents the most promising non‐viral system for CAR‐M engineering, primarily encompassing two major categories: LNPs and liposomes [68]. Among them, LNPs have emerged as the predominant in vivo delivery vectors for CAR‐M construction, owing to their established clinical validation, high nucleic acid delivery efficiency, and highly modular design characteristics [69].

Typical LNPs are composed of ionizable lipids, cholesterol, helper phospholipids, and PEGylated lipids [70]. The ionizable lipid is the critical component determining delivery efficiency. It becomes protonated and positively charged in an acidic environment, forming electrostatic interactions with negatively charged mRNA to drive self‐assembly and facilitating endosomal escape after cellular entry [71]. Cholesterol and helper phospholipids (e.g., DSPC, DOPE) play a role in maintaining the structural stability and membrane fluidity of the nanoparticles [72]. PEGylated lipids prevent particle aggregation by forming a steric hindrance layer, thereby extending circulation time in vivo [73]. This synergistic design of multiple components enables LNPs to efficiently protect mRNA from nuclease degradation and mediate its entry into the cytoplasm for functional expression.

In the context of CAR‐M engineering, LNPs are considered to offer several potential advantages. First, LNPs‐mediated mRNA delivery is a non‐integrating strategy. Based on experience in oncology settings, the mRNA encoding the CAR is transiently expressed in the cytoplasm and naturally degrades; this approach avoids the potential insertion mutation risks associated with viral vectors [74]. Second, the physicochemical properties of LNPs (e.g., particle size, surface charge, component ratios) can be precisely controlled using technologies like microfluidics to optimize their transfection efficiency in macrophages and in vivo distribution [75].

In addition to LNPs, liposomes, as classic lipid carriers, possess a bilayer vesicle structure similar to biological membranes and can encapsulate various therapeutic agents within their aqueous core or lipid bilayer [76]. While generally exhibiting lower gene delivery efficiency compared to LNPs, their mature preparation processes and good biocompatibility retain their utility for co‐delivery of small molecule drugs or combination immunomodulators.

#### 3.1.3 Exosomes and Extracellular Vesicles

Exosomes and extracellular vesicles (EVs) represent natural delivery platforms that have garnered significant attention in recent years. As biologically derived vesicles secreted by cells, exosomes and EVs exhibit low immunogenicity, excellent biocompatibility, and inherent targeting capabilities, making them ideally suited for intercellular communication and biomolecular transport [77]. Their targeting mechanisms involve specific recognition of target cells through membrane‐bound adhesion molecules, integrins, or ligands, with entry primarily occurring via endocytosis [78]. The abundance of specific membrane lipids in exosomes can destabilize the endosomal membrane in acidic endosomal environments, thereby improving delivery efficiency [79]. Furthermore, unlike synthetic nanoparticles, exosomes also possess the ability to interact and fuse with cell membranes, allowing their cargo to bypass endosomal entrapment and be directly released into the cytoplasm [80].

Engineered EVs have already shown promise in enabling CAR‐M‐based tumor immunotherapy. In a notable study, Xiao et al. developed engineered small EVs termed CARmRNA@aCD206 sEVs, which displayed surface‐anchored anti‐CD206 scFv and encapsulated CAR mRNA [81]. Following inhalation, these engineered EVs efficiently accumulated in lung tissue and selectively delivered CAR mRNA to M2 macrophages, enabling in situ CAR‐M generation. This approach suppressed tumor growth and induced long‐lasting immune memory in a murine model of lung metastatic cancer.

Although direct evidence supporting the use of exosomes or EVs to construct CAR‐M for tissue repair applications remains limited, this natural delivery platform is conceptually promising because of its inherent biological properties. For instance, stem cell‐derived exosomes and EVs are intrinsically enriched with various therapeutic bioactive molecules [82]. Through rational engineering, they could be designed to achieve targeted delivery of CAR‐mRNA to macrophages while exerting synergistic therapeutic effects that support tissue repair.

Overall, exosomes and EVs have the potential to serve as natural and engineerable delivery platforms for site‐specific and subpopulation‐selective CAR‐M programming. However, their application in models of tissue injury remains to be systematically evaluated. In addition, purification consistency, scalability, biodistribution, and long‐term safety require further optimization and assessment.

#### 3.1.4 Polymeric Nanocarriers

Synthetic polymer nanoparticles, with their structural diversity, controllable degradability, and ease of functionalization, show great potential for CAR delivery [83]. Biodegradable polymers like poly(lactic‐co‐glycolic acid) (PLGA) and polycaprolactone (PCL) are frequently employed in conjunction with lipid components to encapsulate CAR elements, facilitating stable and sustained gene expression [84]. By modulating polymer molecular weight, comonomer composition, and terminal functional groups, the release kinetics of encapsulated cargo can be finely tuned to meet the specific demands across diverse therapeutic contexts.

Cationic polymer nanoparticles represent a significant class of this type of nanocarriers. They can form polyplexes with negatively charged nucleic acids through electrostatic interactions [85], thereby achieving efficient encapsulation and protection. Upon cellular uptake via endocytosis, these polyplexes primarily rely on the proton sponge effect for endosomal escape [86]. Specifically, the abundant unprotonated amino groups in cationic polymer nanoparticles absorb protons extensively during endosomal acidification, accompanied by chloride ion influx [87]. This process leads to an increase in endosomal osmotic pressure and membrane rupture, ultimately releasing nucleic acids into the cytoplasm [87].

While research directly utilizing cationic polymer nanoparticles to construct CAR‐M for tissue repair is currently limited, their potential in gene delivery for other immune cells has already been demonstrated. For instance, Smith et al. successfully achieved in situ programming of leukemia‐specific T cells using polymer nanoparticles called PBAE‐447. They conjugated T‐cell‐targeting anti‐CD3e f(ab′)2 fragments to the surface of biodegradable poly(β‐amino ester)‐based nanoparticles, which facilitated their selective receptor‐mediated endocytosis by lymphocytes and CAR‐T generation [88]. They also found that the physicochemical properties and transduction efficiency of PBAE‐447 remained unaltered after lyophilization. This significantly simplifies storage requirements and reduces application costs.

Drawing inspiration from this approach, cationic polymer nanoparticles could be surface‐modified with antibodies or ligands targeting macrophage markers (such as CD68, CD206, or CSF1R) to achieve efficient delivery of CAR‐encoding elements to specific macrophage subsets. Future integration of cationic polymer nanoparticles with local controlled‐release systems holds promise for spatiotemporal control of in situ CAR‐M construction, offering a novel strategy that combines materials science and immune engineering advantages for tissue repair and regeneration.

#### 3.1.5 Biomaterial‐Based Scaffolds

**FIGURE 3:**
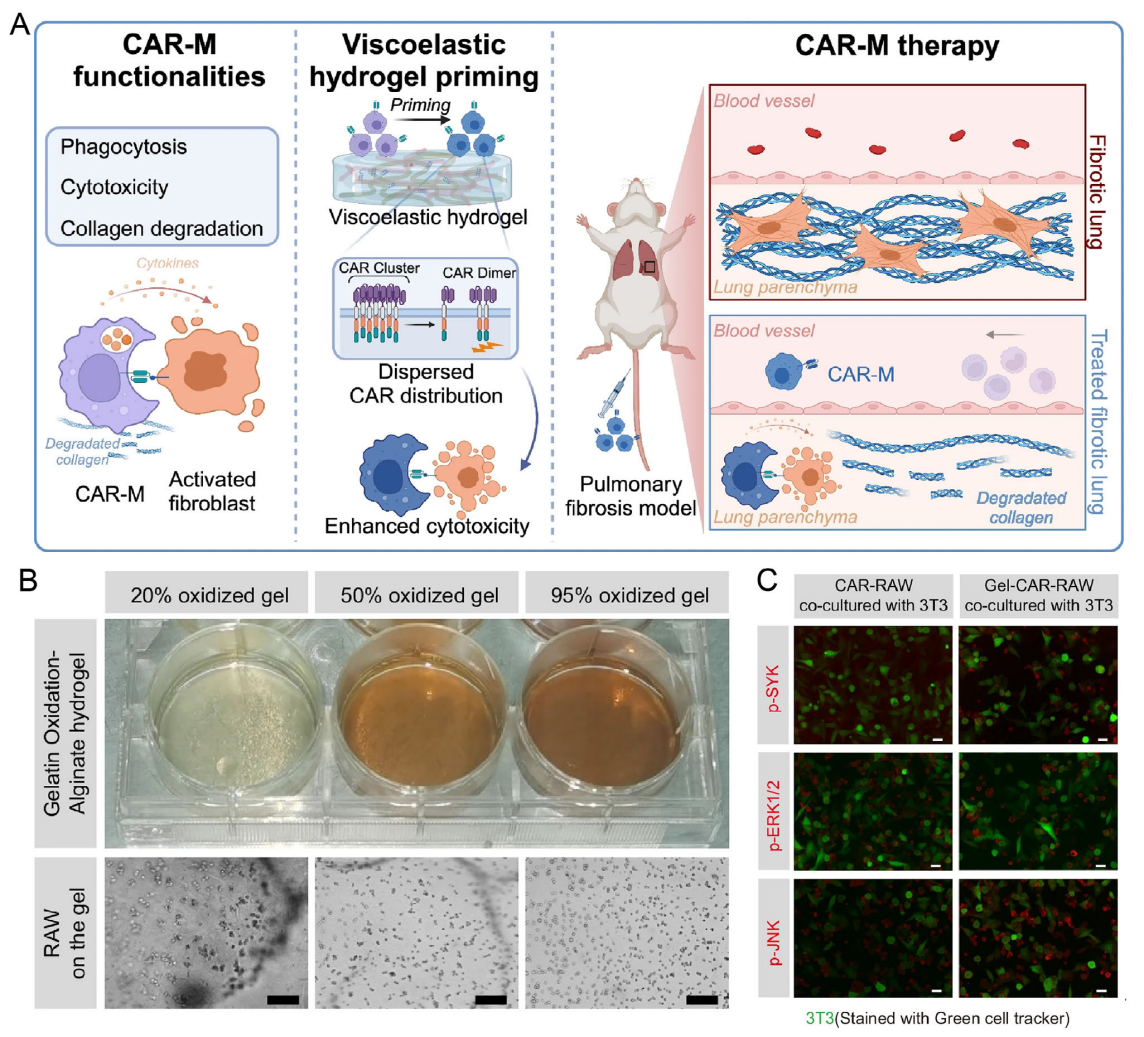
Schematic illustration of viscoelastic hydrogel‐primed CAR‐M therapy for pulmonary fibrosis. (A) Anti‐FAP CAR‐M pretreated with the viscoelastic hydrogel achieved superior therapeutic outcomes, including promoting a dispersed CAR distribution and enhancing their cytotoxic capacity in vitro, and restoring normal lung architecture from fibrotic damage and remodeling the profibrotic immune environment in vivo. (B) Photographs show Gelatin Oxidized‐Alginate hydrogels at varying oxidation levels, with RAW264.7 cells seeded onto their surfaces. Scale bars = 100  µm. (C) Immunofluorescence staining revealed the expression levels of key phosphorylated proteins within the CAR‐related downstream signaling pathway, comparing CAR‐RAW and Gel‐CAR‐RAW cells. Scale bars = 10  µm. Reproduced under the terms of CC BY‐NC‐ND 4.0 license [91]. Copyright 2026, Springer Nature.

Biomaterial‐based scaffolds are fundamental to tissue engineering and regenerative medicine [89], serving not only as structural frameworks but also as potential delivery vehicles for CAR‐M constructs. For example, biodegradable hydrogels and microneedles may function as localized nucleic acid reservoirs, enabling controlled release and macrophage transfection at pathological sites [90]. Beyond passive delivery, biomaterials may also actively modulate CAR‐M function and phenotype through physical mechanical signals. In a recent study on treating pulmonary fibrosis, researchers used a viscoelastic hydrogel to physically precondition CAR‐M and boost their therapeutic efficacy (Figure 3A) [91]. Specifically, they built a viscoelastic hydrogel system based on gelatin and oxidized alginate, maintaining a constant storage modulus of ∼1.3 kPa while modulating the hydrogel's loss modulus and stress‐relaxation rate by varying the alginate oxidation degree (Figure 3B). After 24 h of seeding CAR‐M in the hydrogel, an optimally viscoelastic matrix (50% oxidized alginate) was found to reduce CAR‐M plasma membrane tension and promote CAR dissociation from aggregates into dispersed monomers and dimers, thereby enhancing downstream functional signaling (Figure 3C). In a pulmonary fibrosis mouse model, hydrogel‐preconditioned CAR‐M demonstrated markedly improved cytotoxicity against activated fibroblasts and collagen‐degradation capability, effectively reducing fibrosis degree and improving the immune microenvironment.

Furthermore, through the novel design of scaffold composition and microstructure, it is possible to actively shape a local immune milieu that favors CAR‐M recruitment and function. In sum, biomaterial scaffolds play a multidimensional role. They not only function as efficient, controllable delivery platforms but also serve as essential tools to enhance therapeutic outcomes through biophysical cues and microenvironment modulation.

Table 1 summarizes the key features, advantages, safety concerns, and translational considerations of the five delivery platforms, providing a side‐by‐side comparison to guide platform selection for different tissue‐repair scenarios. Overall, the choice of delivery platform and material design critically dictates the encapsulation efficiency, cellular uptake pathways, endosomal escape capability, and in vivo fate, thereby forming the foundation of efficient and safe CAR‐M programming strategies. Diverse delivery systems exhibit significant variations in their physicochemical properties, functionalization sites, biodegradability, and immunogenicity. Consequently, the selection of a carrier necessitates a systematic evaluation and optimization process that considers therapeutic objectives (e.g., transient controlled expression vs. long‐term stable genetic modification), the route of administration, and the anticipated safety thresholds. For instance, systemic intravenous administration demands carriers with enhanced serum stability, superior targeting specificity, and reduced hepatotoxicity. Conversely, local injection or scaffold‐mediated delivery can leverage the pathological microenvironment for sustained release and spatially precise delivery, imposing less stringent requirements on overall carrier circulation stability but demanding careful consideration of material compatibility with local tissues and the safety of degradation products.

**TABLE 1:** Comparison of delivery platforms for CAR‐M engineering in tissue repair.

Delivery platform	Macrophage‐targeting and transfection ability	Expression duration	Delivery route	Tissue‐repair applicability	Limitations and safety concerns	Translational potential
Viral vectors (AAV, lentivirus)	Generally high for ex vivo transduction; limited direct in vivo targeting and transfection	Long‐lasting, especially lentiviral integration; AAV can support sustained expression	Ex vivo engineering followed by adoptive transfer; rarely direct in vivo delivery	Validated in skin scar prevention, renal fibrosis, and liver fibrosis; more established in oncology than tissue repair	Integration‐related safety concerns for integrating vectors; limited re‐dosing; manufacturing complexity	High for ex vivo CAR‐M manufacture; less attractive for routine tissue‐repair use because of safety and delivery constraints
Lipid‐based platform (LNPs and liposomes)	Engineering‐dependent and can be improved by ionizable lipid design and surface modification	Transient	Primarily in vivo engineering for CAR‐M in situ generation by systemic or local injection; also ex vivo delivery	Validated in liver fibrosis, ischemia‐reperfusion injury, and myocardial infarction injury; strong relevance for in situ CAR‐M generation in tissue repair	Cationic‑induced inflammation; off‑target liver accumulation; potential reactogenicity	Very high, especially for in vivo programming and clinically scalable manufacturing; modular design enables optimization for macrophage targeting
Exosomes and extracellular vesicles	Inherent tropism via surface adhesion molecules and integrins; macrophage selectivity requires further engineering	Typically transient	Mostly in vivo delivery by injection or inhalation; also ex vivo delivery	Conceptually promising platform; direct tissue‑repair evidence currently lacking (validated only in oncology models)	Scalable production and purification remain challenging; batch‐to‐batch consistency; cargo loading efficiency	Promising but still limited by manufacturing and standardization barriers
Polymeric nanocarriers	Moderate; depend on surface functionalization	Tunable (controlled release from days to weeks)	Mostly in vivo or local delivery; often used as hybrid systems with lipids and combinable with scaffolds	Conceptually extrapolated from CAR‑T programming and general macrophage engineering; flexible design and useful for tissue repair	Possible polymer‐related cytotoxicity; inflammatory responses depending on material	Moderate as a flexible engineering platform; requires dedicated CAR‑M validation in tissue‑repair models
Biomaterial‐based scaffolds	Indirect targeting through local retention and spatial control rather than intrinsic macrophage specificity	Sustained local release	Local implantation or injection at injury site	Validated in pulmonary fibrosis; highly relevant for tissue engineering	Material biocompatibility; degradation product safety; surgical and injection‑related risks	High for localized tissue repair; requires further large‑animal and long‑term safety studies

Taking together, the selection of a delivery system for CAR‐M construction is a multi‐objective decision‐making process. Integration of advanced material design, high‐throughput screening technologies, and immunomodulatory principles is expected to yield smarter and tissue microenvironment‐responsive delivery systems, thereby establishing a more robust foundation for CAR‐M applications in precision medicine and tissue engineering.

### 3.2 Functionalization and Targeting Strategies

While macrophages have an inherent ability to uptake particles, they also perform crucial roles in phagocytic clearance, sensing inflammation, and lysosomal degradation [12]. Therefore, when designing delivery vehicles for CAR element delivery to macrophages, it is essential to consider not only efficient cellular uptake but also successful intracellular expression. Among the available delivery systems, lipid‐based delivery systems, particularly LNPs, represent a versatile and tunable platform for in vivo and ex vivo CAR‐M construction due to their clinically validated safety and potent nucleic acid delivery capability. This makes them a critical area for improving CAR‐M therapies. Accordingly, this section focuses on optimization strategies for LNPs, with particular emphasis on ionizable lipids design, surface modification, and physicochemical properties regulation.

#### 3.2.1 Ionizable Lipids Design

Ionizable lipids significantly influence the endosomal escape capability, biodegradation, and immunogenicity of LNPs [92]. Currently, MC3, SM102, and ALC‐0315 are FDA‐approved ionizable lipid components in clinical use [93]. Over the past years, research has indicated that designing lipid molecules for cell delivery, especially immune cells, necessitates a balance between membrane perturbation and cellular tolerance [94]. Although sufficient acid responsiveness and membrane activity can facilitate endosomal escape, excessively strong cationic interactions may cause membrane damage, inflammation activation, and even cellular functional remodeling [95, 96]. These effects could hinder the establishment of a stable and controllable CAR‐M phenotype and therefore require more systematic investigation. Consequently, developing and optimizing ionizable lipids have become a central focus in the engineering design of lipid‐based CAR components delivery systems. Current optimization efforts for ionizable lipid components primarily focus on three major areas: functionalization, structural engineering, and advanced methodologies. Table 2 presents recently developed ionizable lipids with their design principles and applications. Notably, the majority of these innovations were originally designed for cancer immunotherapy, vaccine development, or central nervous system delivery. Their inclusion here reflects that the ionizable lipids design strategy has potential adaptability to CAR‐M‐based tissue‐repair contexts.

**TABLE 2:** Ionizable lipids design and functionalization examples.

Ionizable Lipids	Structure	Design principles	Characteristics and applications
C12‐TLRa [97]	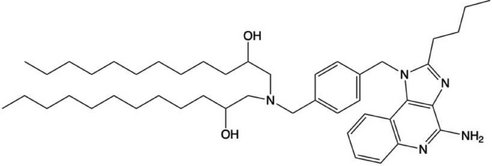	Ring‐opening reaction between amine‐containing TLR7/8 agonist 1 and C12 epoxide	Enhanced intralymphatic dendritic cells activation for mRNA vaccines
C12‐2aN [98]	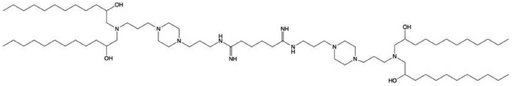	Through imidoester‐crosslinker‐based conjugation chemistry to obtain the crosslinked ionizable lipid	Activating the mTORC2 pathway to stimulate the glycolysis process in dendritic cells and macrophages for mRNA vaccines
a12K4 of PILs platform [99]	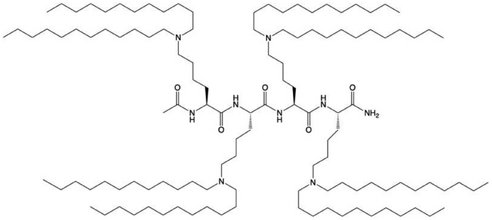	Incorporating artificial ionizable lipids, natural amino acids, and functional molecules into one delivery platform	Tissue‐specific targeting with predictable properties; Kupffer cells and splenic macrophages were transfected by the corresponding LNPs
A2‐B13 [100]	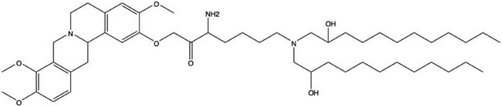	Designing ionizable lipids based on the tetrahydroisoquinoline structure of protoberberine alkaloids	Improving blood‐brain barrier penetration via dopamine D3 receptor‐mediated endocytosis and delivered nucleic acids to astrocytes, neurons, and microglia for brain disease treatment
A5‐B1‐C4.2 [101]	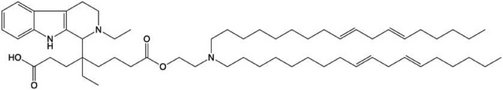	Vinpocetine alkaloid‐inspired cyclic tertiary amine compounds design	Self‐enhanced brain‐targeted nucleic acid delivery ability for brain disease treatment
NT1‐O12B [102]	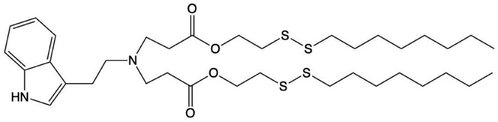	Neurotransmitter‐derived ionizable lipids through Michael addition reaction between the primary amine in the neurotransmitter and alkyl acrylate	Carrying various drugs to cross the blood‐brain barrier and treating central nervous system diseases
SSC6F5 [103]	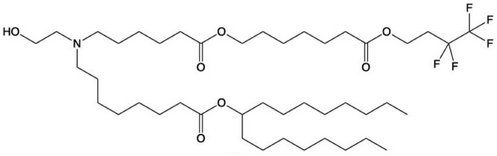	Varying hydrophobic tail architectures and fluorine stoichiometry based on the clinically approved SM‐102 and ALC‐0315 scaffold	Spleen‐targeted (including splenic macrophages, dendritic cells, and B cells) mRNA delivery for cancer immunotherapy
A1F5C5 [104]	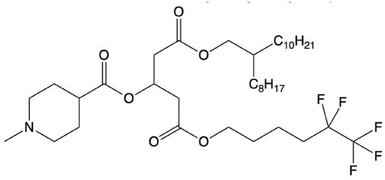	Using a combinatorial chemistry approach to synthesize a library of fluorinated ionizable amino lipids	CAR‐M therapy in solid tumor; enhanced anti‐PD‐L1 immunotherapy
1d [105]	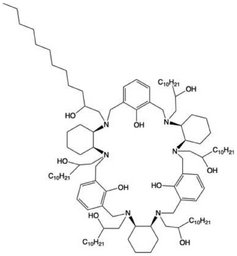	Macrocyclic chemistry‐based rigid Calixsalan macrocyclic core to enhance structural stability to the LNPs and prevent premature payload leakage in the circulation	Macrophage‐targeted Trem2 siRNA delivery to reduced plaque burden and treat atherosclerosis
MeDZ [106]	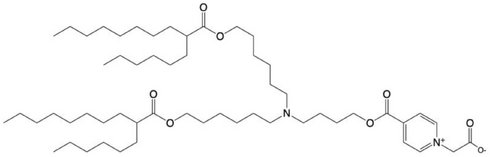	Combining a pyridine‐based carboxybetaine zwitterionic headgroup, biodegradable multitailed alkyl chains and a tertiary amine linker to achieve efficient endosomal escape and low‐inflammatory reactogenicity	Incorporated MeDZ lipids into the commercially available BNT162b2 LNPs from Pfizer‐BioNTech to obtain a new efficient mRNA vaccine

(1) At the functionalization level, by incorporating modules with specific biological activity into lipid structures, LNPs can transform from simple delivery vehicles into active participants in therapeutic process. For instance, in the realm of immune activation, Michael J. Mitchell's team has conducted a series of studies. They substituted a portion of the ionizable lipid component of LNPs with an adjuvant lipidoid designed based on TLR7/8 (Toll‐like receptor agonists) [97]. This method significantly enhances the activation of dendritic cells and the immune response in mice inoculated with the SARS‐CoV‐2 mRNA‐LNP vaccine. In their most recent research, the team further utilized imidoester‐based conjugation chemistry to construct a new crosslinked ionizable lipid called C12‐2aN [98]. The LNPs constructed based on C12‐2aN not only achieve efficient mRNA expression but also boost glycolysis in macrophages and monocytes. This strategy, which integrates mRNA delivery with immune cell metabolic reprogramming into a single LNP formulation, offers valuable insights for the development of next‐generation mRNA vaccines. Similarly, future research could design an ionizable lipid component capable of delivering CAR mRNA to macrophages while modulating their metabolic preference toward OXPHOS. This would promote macrophages polarization toward an M2 phenotype with tissue‐repair functions at inflammatory sites.

**FIGURE 4:**
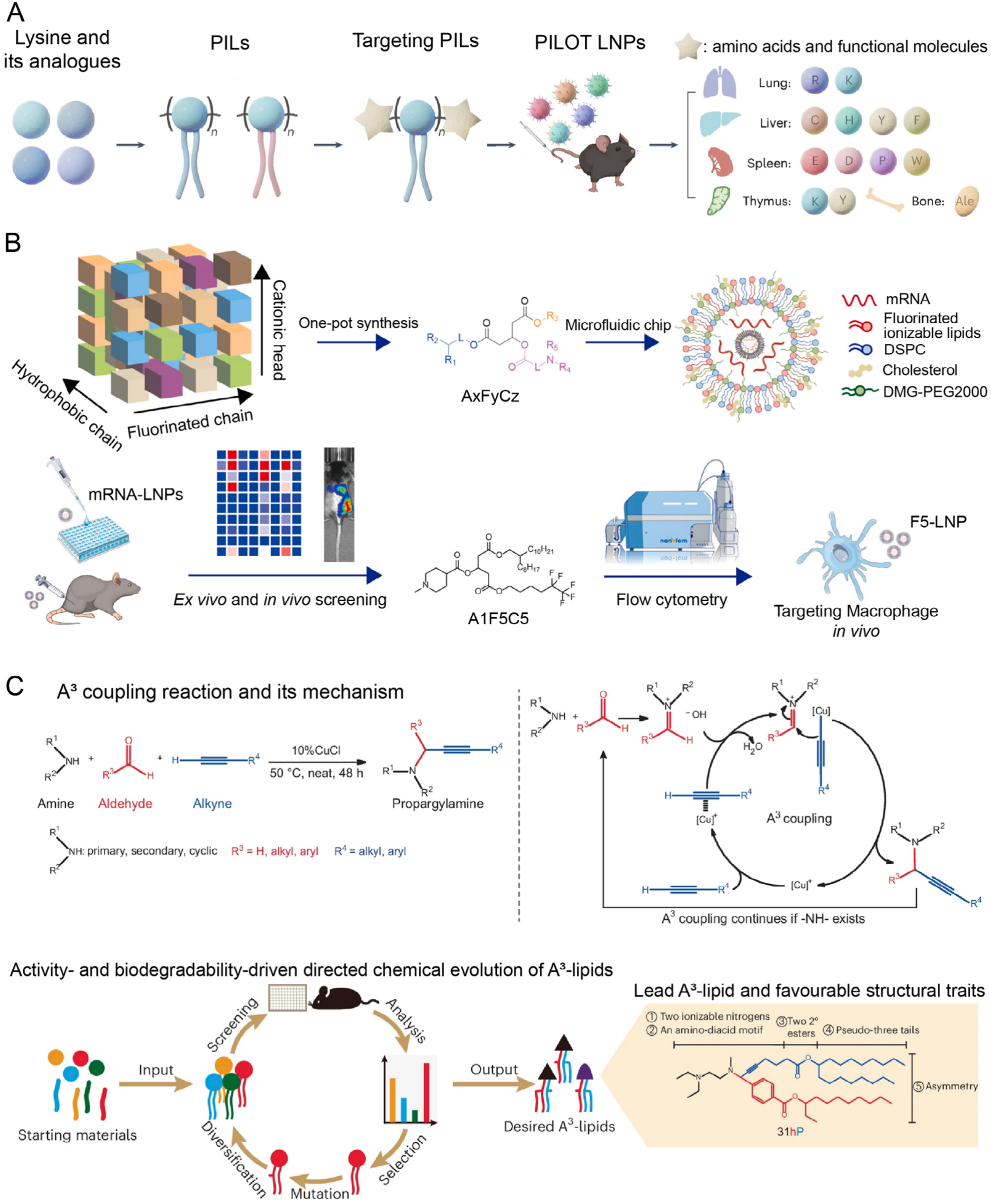
Functionalization strategies of ionizable lipids. (A) Construction of PIL library and PIL‐based mRNA‐LNPs. PILs are composed of ionizable lipid building blocks and diverse amino acids or functional molecules. PILOT means PIL‐driven organ targeting platform, which enables organ‐specific and organ‐tunable mRNA delivery. Reproduced with permission [99]. Copyright 2026, Springer Nature. (B) The creation of a fluorinated lipid library, coupled with the synthesis of LNPs and subsequent cell‐ and animal‐level screenings, led to the identification of an optimal fluorine‐containing lipid. Reproduced with permission [104]. Copyright 2026, Elsevier. (C) A3‐coupling reaction and activity‐ and degradability‐driven directed chemical evolution of A^3^‐lipids. Reproduced with permission [107]. Copyright 2024, Springer Nature.

In the aspect of organ targeting, Cheng and Wei's group merged two classes of biomaterials, including peptides with variable structures in biology and ionizable lipids in lipid nanotechnology, to create a novel Peptide Ionizable Lipid (PIL). As shown in Figure 4A, they subsequently developed an organ‐targeting mRNA delivery platform named PILOT (Peptide Ionizable Lipid‐driven Organ Targeting) [99]. The PILOT platform could achieve precise organ targeting without ligand modification by incorporating different amino acids. After treatment with the corresponding PILOT LNPs, tdTomato fluorescence signals could be detected in Kupffer cells (hepatic resident macrophages) or in splenic macrophages. Extrapolated from these findings, similar lipid‐structure‐driven organ tropism could potentially be leveraged to generate CAR‐M in specific injured organs. However, this premise requires further validation.

Beyond organ targeting, recent studies have seen the emergence of ionizable lipids with unique bio‐inspired structures, such as A5‑B1‑C4.2, NT1‑O12B, and A2‑B13, designed based on vinpocetine, neurotransmitter‐mimicking lipids, and berberine‐inspired molecular scaffolds, respectively [100, 101, 102]. These ionizable lipids enable the successful delivery of mRNA or other cargo to the brain parenchyma via intravenous injection, representing a breakthrough in overcoming the delivery challenges of breaching the blood‐brain barrier. Although these applications are not directly about CAR‐macrophage generation, the approach of designing bio‐inspired ionizable lipids according to the tissue injury microenvironment is worth learning from.

(2) In terms of structural engineering, fluorination modification for ionizable lipids stands out as a particularly prominent strategy. Fluorinated lipid tails have the unique characteristics of fluorocarbon chains, such as high electronegativity, low polarizability, and simultaneous hydrophobicity/lipophobicity [108]. They not only improve membrane fusion kinetics and increase nanoparticle mobility for enhanced endosomal escape but also enable precise organ targeting. Building upon the clinically approved SM‐102/ALC‐0315 ionizable lipid scaffold, Liu's team designed a fluorinated ionizable lipid (SSC6F5) by rationally adjusting the hydrophobic tail and fluorine stoichiometry [103]. Their investigation found that SSC6F5‐based LNPs achieved over 90% spleen targeting specificity, with mRNA transfection levels 10.6‐fold higher than that of the clinical SM102/18PA LNPs. Furthermore, it efficiently transfected splenic macrophages, presenting a potent tool for in situ macrophage engineering. Concurrently, other researchers employed a combinatorial chemistry approach to synthesize a library of fluorinated ionizable amino lipids (Figure 4B) [104]. Through rigorous screening, they identified the candidate compound A1F5C5, whose specific configuration with five fluorine atoms, uniquely conferred superior membrane fusion capability. Following intravenous administration, A1F5C5‐based LNPs achieved selective mRNA delivery to splenic macrophages and tumor‐infiltrating macrophages. While fluorination modification has been demonstrated to confer tissue tropism and macrophage targeting, the reported functional readouts have been limited to antitumor therapy. Their direct applications in tissue‑damage models have yet to be reported; thus, their potential remains speculative at this stage.

Besides fluorination modification, the acid dissociation constant (pKa) of lipid headgroups is also identified as critical for achieving efficient endosomal escape and high in vivo potency [109]. Hassett et al. showed that, for mRNA vaccine applications in terms of H10‐specific IgG expression, the optimal pKa range for intramuscular delivery is 6.6–6.9 [110]. Chen et al. found that LNPs containing an ionizable lipid with a thioamide adjacent to the tertiary amine have a lower pKa than those containing an analogous lipid with a conventional amide, and they exhibit significantly improved mRNA transfection efficiency both in vitro and in vivo [111].

Tail‐chain unsaturation also influences mRNA transfection efficiency [112]. Liu's group reported that, for ionizable lipids with identical backbones, higher total degrees of unsaturation (Ω) in the lipid tails correlate with higher mRNA encapsulation efficiency (85%–95%). Using interleukin‐27 (IL‐27)‐encoded mRNA as a model, when Ω ≥ 2, LNPs show superior endosomal escape and transfection efficiency in HepG2 cells. In murine experiments, this system achieves efficient delivery and expression while maintaining low immunogenicity and inflammatory responses [113]. These findings in hepatocytes could potentially guide the optimization of ionizable lipid tail‐chain structures for macrophage transfection, and cross‐cell‐type validation is required.

(3) In recent studies, advanced methodologies are reshaping the development paradigm for ionizable lipids [92]. Han et al. have developed a directed‐evolution platform based on the amine–aldehyde–alkyne (A3) coupling reaction. This A3 platform enables iterative optimization of lipids from mutagenesis to screening within weeks to months and rapidly identified novel lipids whose delivery efficiency and biodegradability rival or surpass clinical controls (Figure 4C) [107]. Meanwhile, machine learning‐assisted design is becoming a key tool in lipid research and development [114]. By building artificial Intelligence (AI) models that learn the structure–delivery efficiency–toxicity relationships, virtual screening and rational prediction of new lipids can be realized. This significantly reduces the trial‐and‐error costs in development. Moreover, integrating combinatorial library construction with high‐throughput screening enables the generation of candidate libraries comprising hundreds to thousands of structurally diverse lipids. Preferred molecules can then be rapidly identified through in vitro and in vivo functional evaluations [115].

To sum up, direct investigations into ionizable lipid optimization specifically for CAR‑M construction remain limited. Nevertheless, strategies developed for nucleic acid delivery to dendritic cells and other immune cells, as well as their applications in anti‐tumor therapy and mRNA vaccines, may offer valuable guidance for improving macrophage compatibility and advancing CAR‑M engineering for tissue repair.

#### 3.2.2 Surface Modification

Beyond optimizing ionizable lipid components, direct surface modification of LNPs remains a key strategy to enhance carrier performance and impart smart‐responsive behaviors. Although there are currently few studies on surface‐modifying LNPs for improving CAR‐M engineering and tissue repair, given that macrophages express diverse specific receptors, precise targeting of particular macrophage subsets could be achieved via receptor/ligand‐mediated interactions. For instance, incorporating functional ligands such as mannose or hyaluronic acid onto the LNP surface enables interaction with receptors like CD206 or CD44, respectively, thereby increasing the selective delivery of the carrier to macrophages [116]. In such designs, the type of ligand, conjugation method, surface density, and the length and rigidity of linkers all significantly influence targeting efficiency and in vivo behavior [117]. Excessive ligand density may lead to particle aggregation, non‐specific binding, or accelerated clearance, while insufficient density can diminish targeting capability. Therefore, establishing a balance between targeting specificity and circulatory stability by integrating chemical design with experimental screening to optimize ligand presentation is a key point for the precise delivery of CAR‐encoding elements.

**FIGURE 5:**
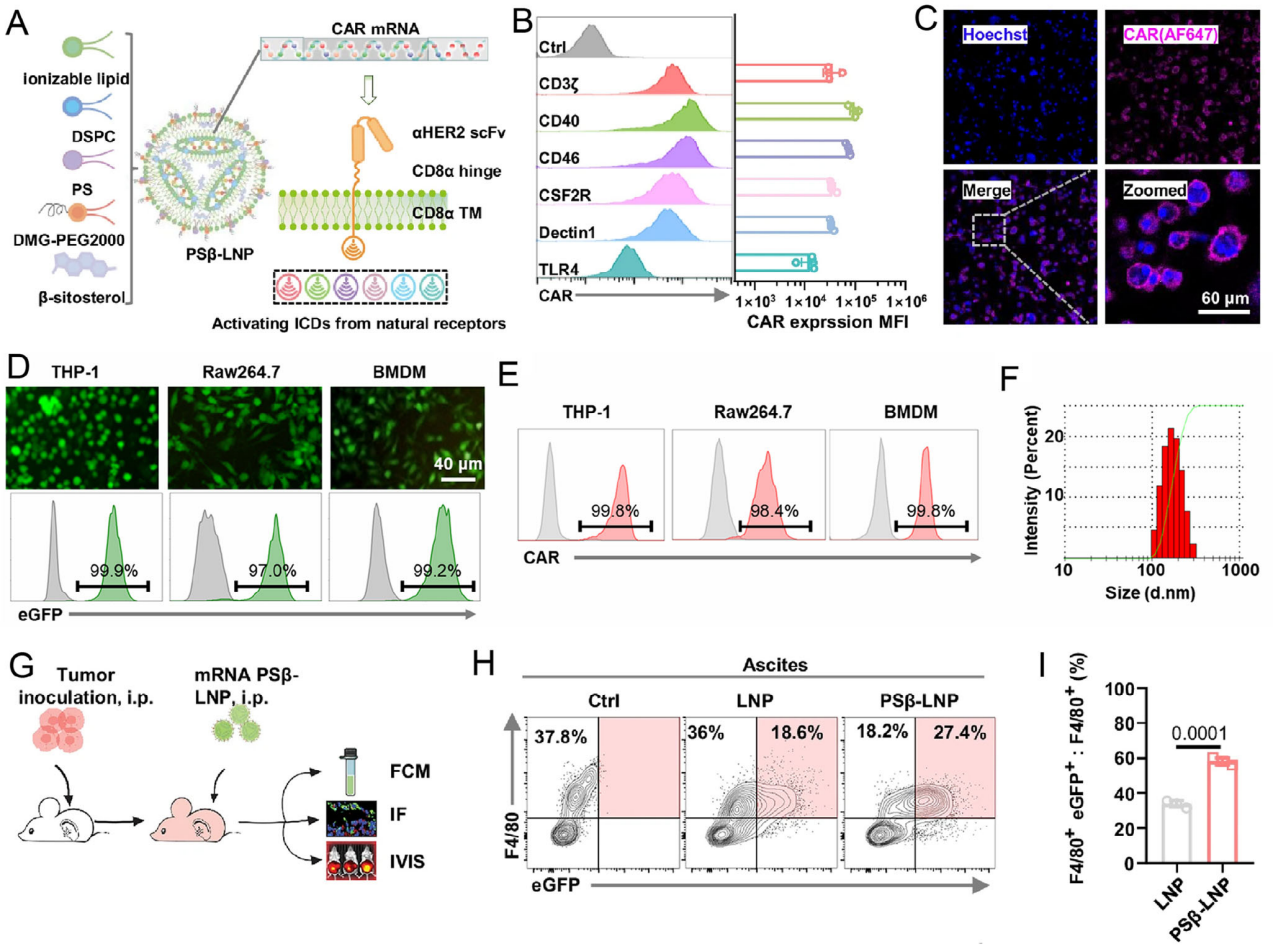
Intraperitoneal CAR‐M engineering via modified LNPs for cancer immunotherapy. (A) Schematic illustration of CAR‐M generation using mRNA‐loaded PSβ‐LNP. (B) CAR expression on BMDMs at 24 h by flow cytometry (B) and confocal imaging (C). (D) eGFP‐mRNA PSβ‐LNP transfection efficiency in different macrophages. (E) CD3ζ CAR‐mRNA PSβ‐LNP transfection efficiency in different macrophages. (F) The size distribution of the PSβ‐LNP. (G) Schematic Illustration of in vivo verification experiments. (H) eGFP expression status in ascites cells after administration of eGFP‐mRNA LNP. (I) eGFP^+^ and F4/80^+^ proportion among F4/80^+^ cells. Reproduced under terms of CC BY‐NC‐ND 4.0 license [37]. Copyright 2025, Springer Nature.

Besides receptor/ligand‐mediated targeting, antibodies, nanobodies, polypeptides, and small molecules can serve as surface modification units to improve the cellular selectivity of LNPs. For instance, utilizing click chemistry to conjugate anti‐CD3 or anti‐Ly6c monoclonal antibodies to the termini of PEGylated lipids has been used to explore active targeting of LNPs to specific immune cell subsets, which may inform the design of macrophage‐selective systems for CAR‐M programming [118]. Furthermore, to address the challenges of the in vivo delivery of CAR‐encoding elements to macrophages, Tan's group engineered the macrophage‐targeting LNPs by integrating phosphatidylserine (a macrophage‐specific uptake unit) and β‐sitosterol (an enhancer of mRNA transfection efficiency) into a conventional LNP formulation to construct the PSβ‐LNP delivery system (Figure 5A,F). This system demonstrated in vitro transfection efficiency exceeding 99% across various types of macrophages (Figure 5B–E). Following a single intraperitoneal injection of PSβ‐LNPs loaded with eGFP‐mRNA in mice (colon cancer CT26 syngeneic model with peritoneal metastasis), macrophages comprised approximately 45% of the total peritoneal cell population, with over 60% of these macrophages expressing eGFP at 18 h, indicating significantly higher transfection efficiency compared to conventional LNPs (Figure 5G–I) [37]. Notably, while this study provides a proof‐of‐concept for macrophage‐targeted mRNA delivery and CAR expression, its direct applicability to tissue‐repair settings requires further validation.

For central nervous system diseases, Yan et al. employed cell‐penetrating peptide (CPP)‐modified LNPs in an intranasal delivery model to enhance their ability to cross the nasal epithelial barrier, and further conjugated the MG1 peptide to enable precise CAR delivery to microglia [40]. In the APP/PS1 mice model (Alzheimer's disease model), in situ generated CAR‑M significantly reduced β‐amyloid plaque deposition in the brain, suppressed neuroinflammatory responses, and improved cognitive functions. In addition, biomimetic approaches, such as coating nanoparticles with cell membranes, have also been explored to improve the carriers’ biocompatibility, immune evasion, and inherent targeting. One recent study has developed a CAR‐encoding plasmid delivery system using enucleated mesenchymal stem cells (MSCs). This system leverages MSCs’ innate homing ability for targeted delivery and in vivo construction of CAR‐M in a glioblastoma model [119]. However, its applicability to tissue repair awaits experimental confirmation.

Collectively, surface modification of LNPs represents a critical strategy for constructing efficient and specific CAR‐M programming platforms. Nevertheless, in tissue repair‐related verification, this field remains underdeveloped, and future investigations should address this gap. Efforts may focus on developing multifunctional integrated carriers that combine targeted delivery, immune regulation, and real‑time monitoring for precise control of CAR‑M generation.

#### 3.2.3 Physicochemical Properties Optimization

**FIGURE 6:**
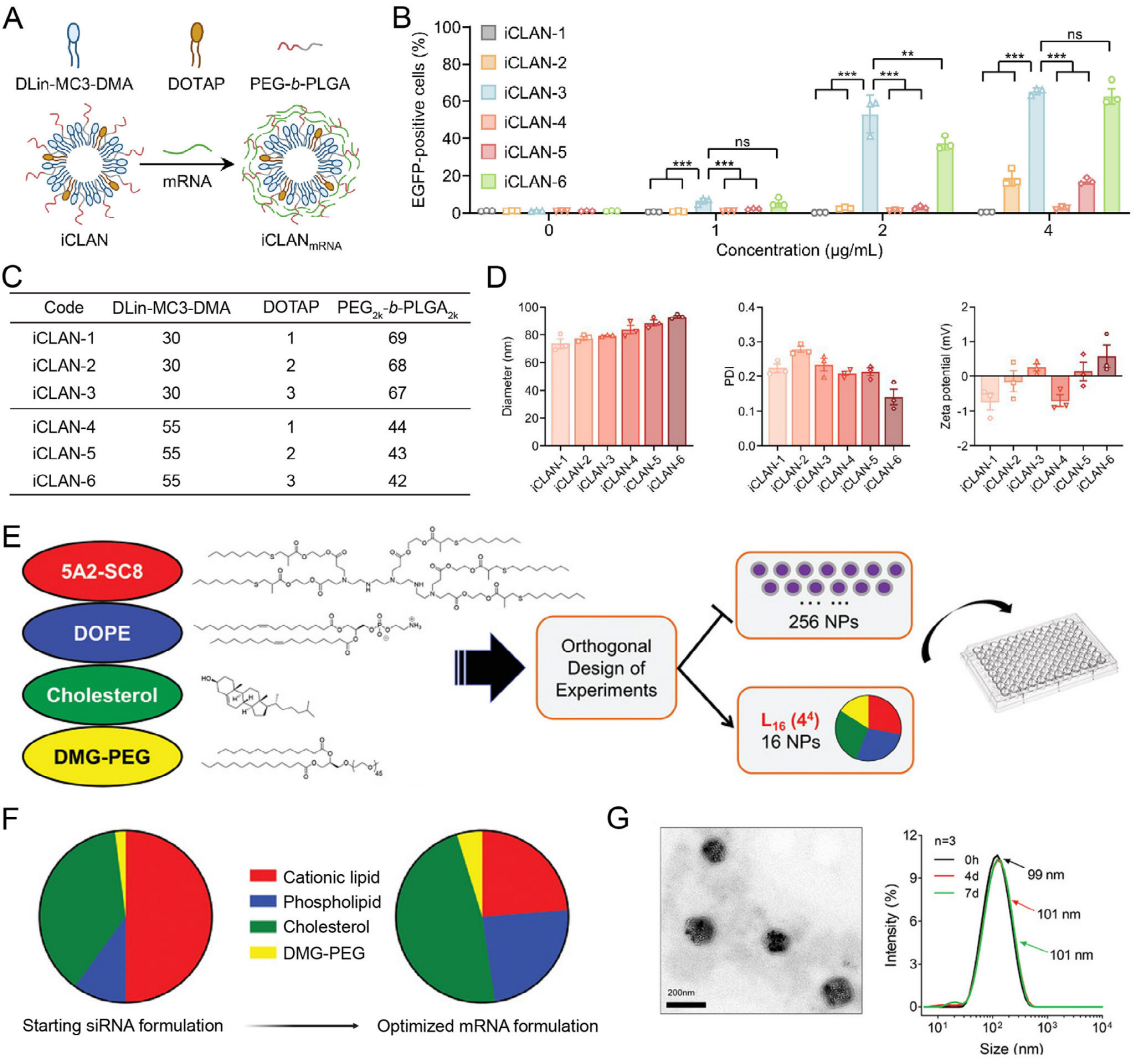
Optimization and characterization of LNP physicochemical properties for enhanced mRNA delivery. (A) Schematic illustration of iCLAN design for CAR‐M generation used in cancer immunotherapy. (B) EGFP‑positive BMDM percentages after transfection with different iCLAN formulations. (C) Table detailing the weight percentages of DLin‑MC3‑DMA, DOTAP, and PEG‑*b*‑PLGA in each iCLAN formulation. (D) Physicochemical properties of different iCLAN carriers, including hydrodynamic diameter, PDI, and zeta potential. Reproduced with permission [122]. Copyright 2026, American Chemical Society. (E) Schematic illustration of orthogonal experimental design used to systematically optimize dendrimer‑based LNP delivery systems. (F) Comparison of molar ratios of cationic lipid, phospholipid, cholesterol, and DMG‑PEG between the starting siRNA formulation and the optimized mRNA formulation. (G) Representative TEM image (left) and stability measurements (right) of the optimized LNPs. Scale bars = 200 nm. Reproduced with permission [123]. Copyright 2018, Wiley‐VCH.

The physicochemical properties of LNPs, including particle size, size distribution, and surface charge, are largely governed by their formulation composition and directly influence cellular uptake, biodistribution, and therapeutic efficacy [116, 120, 121]. Even when the same classes of lipid components are employed, varying their ratios can alter these properties and consequently affect transfection performance. For instance, Sun et al. developed a macrophage‑preferential delivery system, termed iCLAN, which enables stable CAR‑M generation (Figure 6A) [122]. By simply adjusting the weight percentages of DOTAP, DLin‐MC3‐DMA, and PEG‐*b*‐PLGA, the resulting nanoparticles exhibited distinct variations in particle size, polydispersity index (PDI), and zeta potential (Figure 6C,D). These changes correlated with transfection efficiency of EGFP reporter mRNA in BMDMs (Figure 6B). The optimal formulation, iCLAN‐3, achieved the highest EGFP‐positive cell percentage and showed effective CAR‐M production, as well as marked antitumor efficacy in vivo. These findings confirm that formulation optimization can reshape LNP physicochemical properties and thereby influence CAR‑M engineering outcomes.

A similar optimization strategy has also been applied in other delivery contexts. Cheng et al. systematically optimized dendrimer‑based LNPs for mRNA delivery (Figure 6E) [123]. They found that reducing the ionizable cationic lipid from ∼50% to ∼24%, increasing DOPE to ∼24%, and raising DMG‑PEG to ∼4.76% yielded stable LNPs with a size of ∼100 nm and near‑neutral charge (Figure 6F,G). The LNPs with this optimized formulation achieved over 44% hepatocyte transfection efficiency in vivo and effectively restored liver function in a tyrosinemia type I mouse model. Although these studies were not conducted directly in the context of CAR‑M for tissue repair, together, they highlight that optimizing LNP physicochemical properties, whether through ratio adjustment or component selection, is a versatile strategy to enhance delivery efficiency.

Overall, lipid‐based platforms exhibit important advantages for CAR components delivery and CAR‐M generation, stemming not only from their high nucleic acid delivery efficiency but also from their highly modular and engineerable nature. Notably, the synergistic interplay of three key strategies for LNPs engineering, novel lipid molecule design, surface modification, and formulation optimization (i.e., physicochemical property regulation), collectively dictates the carriers' intracellular fate and ultimate functions. Future development of lipid‐based platforms is likely to focus on: (1) developing ionizable lipids better suited for the intracellular environment of macrophages; (2) constructing surface targeting modules with in vivo subset selectivity; (3) enhancing endosomal escape while mitigating inflammatory activation; and (4) exploring their potential integration with tissue engineering contexts for localized and precise delivery to necrotic or fibrotic tissues in injury sites.

### 3.3 Interaction With Macrophage Behaviors

The interaction between delivery systems and macrophage behaviors is critical for the successful application of CAR‐M therapy, as it dictates the intracellular trafficking fate of nucleic acid payloads, the efficiency of CAR expression, and the ultimate functional state of the macrophages. Consequently, this section will delve into the uptake pathways and intracellular fate, as well as the impact of carrier chemistry on macrophage polarization, to foster a deeper understanding.

#### 3.3.1 Influence of Uptake Pathways and Intracellular Trafficking on CAR Expression

Macrophages, as professional phagocytes, possess multiple pathways for internalizing exogenous substances, including phagocytosis, receptor‐mediated endocytosis (e.g., clathrin‐ and caveolin‐mediated pathways), and macropinocytosis [124, 125, 126]. These distinct uptake routes lead to different intracellular transport pathways, which directly impact whether nucleic acid payloads can effectively escape lysosomal degradation and achieve functional expression [127]. First, the particle size of carriers significantly influences their cellular entry mechanisms [128]. Generally, nanoparticles smaller than 200 nm rely on endocytosis, while larger particles (>500 nm) are more readily recognized and taken up by macrophages through phagocytosis [129, 130]. This implies that for small nucleic acid carriers, endosomal escape efficiency often becomes a critical bottleneck limiting CAR expression. While for larger particles, the subsequent intracellular processing often affects payload release and expression duration.

Moreover, CAR intracellular trafficking mechanisms mediated by different vector types exhibit variability, as mentioned in Section 3.1. In tissue engineering scenarios, the intracellular behavior of the carrier needs to be matched with the therapeutic objectives. For acute inflammatory interventions that require rapid initiation of CAR expression and short‐term immune reprogramming, LNP systems are more advantageous due to their fast expression kinetics and strong time sensitivity. For chronic tissue damage or localized immune regulation that requires maintaining functional output over longer periods, viral vectors or biodegradable polymer systems may be more suitable. Thus, the choice of uptake pathway and intracellular trafficking route fundamentally determines whether the CAR payload can be converted into effective functional output in the target cells.

#### 3.3.2 Effects of Carrier Chemistry on CAR‐M Polarization and Immune Function

Macrophages have high plasticity and can dynamically switch between M1 pro‐inflammatory and M2 anti‐inflammatory (pro‐repair) phenotypes in response to different stimuli [24]. This means that the therapeutic efficacy of CAR‐M depends not only on the design of the CAR molecule, but is also significantly modulated by the chemical properties of the carriers. Factors such as surface charge, degradation profile, and components may serve as potential invisible cues that participate in the fate regulation of macrophages [131].

Among these, surface charge is an important chemical parameter [132]. Based on extensive studies in general macrophage biology, cationic carriers are generally considered to interact more readily with the cell membrane, thereby potentially enhancing uptake efficiency. However, excessive cationic character may potentially elicit inflammatory responses and promote the release of pro‐inflammatory cytokines. By contrast, neutral or slightly anionic carriers often exhibit better biocompatibility and lower immunotoxicity, although their cellular uptake efficiency may be relatively limited. Therefore, carrier charge design needs to be balanced according to the specific application scenarios.

In addition to charge effects, carrier degradation products can also influence macrophage polarization. For instance, lactic acid is a degradation product of PLGA [133]. Lactic acid can be metabolized by macrophages and then promotes M2 polarization by OXPHOS enhancement [134, 135]. This could offer synergistic benefits in tissue repair requiring an anti‐inflammatory milieu. Conversely, the degradation products of certain cationic polymers may activate TLR signaling pathways, inducing M1 polarization [136, 137]. This highlights the importance of considering the long‐term impact of degradation products on the local immune microenvironment, beyond just delivery performance, when selecting nanocarriers or tissue engineering scaffolds, in order to avoid conflicting with CAR‐M therapeutic objectives.

Further, carrier components and functionalized modifications may not only play roles in targeted delivery, but may also directly participate in immune regulation. For instance, certain ligands, proteins, or biomimetic membrane components may enhance cell recognition and simultaneously influence macrophage activation state and phagocytic behavior through receptor‐mediated signaling [138]. Similarly, bioactive lipids or metabolically active components embedded within the carrier structure may also exert immune‐modulating effects during delivery, enabling an integrated delivery‐regulation design [139]. This concept is particularly important for non‐oncologic diseases, because in treating tissue damage, chronic inflammation, and fibrosis, the intrinsic biological properties of the carrier can amplify or counteract the therapeutic effects of CAR‐M.

In conclusion, CAR design and carrier chemistry collaboratively shape the polarization state and functions of engineered macrophages. During CAR‐M programming, comprehensive consideration must be given to the immunogenicity of carrier materials, the signaling roles of targeting ligands, and the biological effects of degradation products. A profound understanding and judicious utilization of these interaction dynamics will provide crucial underpinnings for achieving precise, efficient, and safe CAR‐M applications in tissue engineering.

## 4 CAR‐M‐Based Therapy in Tissue Engineering and Regenerative Medicine

Traditionally, CAR‐M research has focused primarily on tumor immunotherapy. However, macrophages are also central regulators of tissue homeostasis, injury response, and repair/remodeling. Consequently, researchers are extending CAR‐M technology into tissue engineering and regenerative medicine, endowing it with specific recognition–clearance–remodeling functions to enable active intervention in diseased or injured tissues. After systematically reviewing the delivery systems that may be suitable for CAR‐M engineering and strategies for their design optimization and functionalization, this section highlights the advances in CAR‐M applications for liver tissue engineering and cardiovascular diseases, aiming to provide paradigms applicable to the regeneration and repair of other tissues.

### 4.1 CAR‐M in Liver Repair

The liver serves as the body's central organ for metabolism, detoxification, and immune regulation [140]. However, multiple factors including alcohol abuse, hepatitis virus infection, drug‐induced injury, and obesity can trigger hepatocellular injury, inflammatory infiltration, and the onset of fibrosis [141]. Liver fibrosis is a common pathological feature of chronic liver disease progression, characterized by aberrant activation of hepatic stellate cells (HSCs), which leads to excessive ECM deposition and normal hepatic architecture disruption [142]. Without promptly addressing, this process may ultimately progress to cirrhosis or hepatocellular carcinoma [143]. Current treatments for liver fibrosis and chronic liver diseases mainly rely on etiological control (e.g., antiviral therapy, alcohol abstinence) and symptomatic supportive care [144]. To date, there is still no effective drug to reverse established liver fibrosis or even early cirrhosis [145]. Liver transplantation is a curative approach for end‐stage liver disease, but it is limited by donor shortages, immune rejection, and high costs [146].

Macrophages play a central regulatory role in maintaining hepatic homeostasis, modulating inflammation, and reversing fibrosis [147]. Notably, the liver intrinsically hosts resident macrophages known as Kupffer cells [148]. Emerging CAR technology offers engineering strategies to endow macrophages with the ability to specifically recognize and clear pathogenic cells and excessive ECM [82]. This approach thus holds great promise as a precision macrophage‐based immunotherapy strategy for liver regenerative medicine.

**FIGURE 7:**
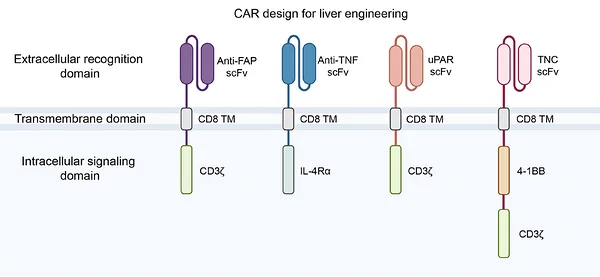
Validated CAR design for macrophages engineering in liver repair. Anti‐FAP scFv targets activated hepatic stellate cells (HSCs). Anti‐TNF scFv recognizes inflammation. uPAR is a cell surface protein that is overexpressed in liver fibrosis. Anti‐TNC scFv makes CAR‐M target dense ECM in the liver fibrosis region. Created in BioRender. Cui, S. (2026) https://BioRender.com/co780cc.

As summarized in Table 3, CAR‑M has been applied to a range of liver disease models, including CCl_4_‑induced fibrosis, metabolic‑associated liver fibrosis, and ischemia‑reperfusion injury. Figure 7 illustrates the CAR designs used in these studies. Across these studies, several common themes emerge. First, the majority of current CAR‑M approaches for liver repair rely on ex vivo engineering via viral vectors (adenoviral or lentiviral) followed by adoptive transfer, although recent work has demonstrated the feasibility of direct in vivo CAR‑M generation using ROS‑responsive LNPs [57]. Second, the target antigens have expanded from activated HSCs markers (uPAR and FAP) to ECM components (TNC) and inflammatory signals (TNF), reflecting a growing recognition that effective liver repair may require addressing both cellular and microenvironmental drivers of fibrosis. Third, therapeutic readouts consistently show reduction in fibrosis, improved liver function, and, in some cases, recruitment of endogenous immune cells that amplify anti‑fibrotic responses. Finally, safety considerations, particularly long‑term persistence and off‑target effects, remain incompletely characterized, highlighting the need for further preclinical evaluation.

**TABLE 3:** Representative CAR‐M applications in liver repair.

Disease model	Target cell or antigen	Macrophage type	Administration route	Delivery method	Therapeutic readout	Unresolved safety issue
Murine models of CCl_4_‐induced liver fibrosis and cirrhosis; DDC diet‐induced cholestatic fibrosis; and HFCF diet‐induced NASH fibrosis model [43]	uPAR^+^ HSCs	Mouse BMDMs	Intravenous injection (1×10^6^ cells/mouse)	Adenoviral transduction ex vivo	Reduced disease‐associated uPAR^+^ cells; Reduced liver fibrosis and cirrhosis level; Remodeled the immune microenvironment and triggered a T‐cell response that amplifies anti‐fibrotic immunity	Long‐term safety
Murine models of CCl_4_‐induced liver fibrosis and HFMCD diet‐induced metabolic‐associated liver fibrosis [57]	FAP^+^ HSCs	Endogenous fibrosis‐associated macrophages	Intravenous injection (LNP dosage: 1 mg/kg twice a week)	ROS‐responsive LNP‐mediated in vivo dual mRNA delivery (CAR and TRIM13)	Enhanced clearance of FAP^+^ activated HSCs; Induced an anti‐inflammatory macrophage phenotype and alleviated hepatic inflammation; Restored liver function and elicited adaptive antifibrotic immunity	Potential tissue injury caused by off‐target anti‐FAP CAR expression.
Murine models of CCl_4_‐induced liver fibrosis and MCD diet‐induced NASH fibrosis [149]	TNC (Tenascin‐C, an ECM protein)	Mouse BMDMs	Intravenous injection (2×10^6^ cells/mouse)	Lentiviral transduction ex vivo	Reduced TNC protein levels, suppressed HSCs activation and ECM deposition; Improved liver function and prolonged survival in mice with cirrhosis; Recruited M2‐polarized macrophages and CD8^+^ T cells to liver	Long‐term safety, off‐target ECM recognition, and systemic immune effects
Liver ischaemia reperfusion injury mouse model [42]	Tumor necrosis factor (TNF, an inflammatory factor)	Mouse BMDMs	Intravenous injection (2×10^6^ cells/mouse)	Lentiviral transduction ex vivo	Reduced hepatocyte necrosis and inflammation; Signal switching strategy: treating a broad spectrum of acute and chronic inflammatory‐related diseases	Potential unintended immunosuppression in non‐target TNF‐rich inflammatory tissues

Abbreviations: CCl4: carbon tetrachloride; DDC: 3,5‐dietthoxycarbonyl‐1,4‐dihydrocollidine; FAP: fibroblast activation protein; HFCF: high‐fat/cholesterol/fructose; HFMCD: methionine‐choline‐deficient; IL‐4Rα: interleukin‐4 receptor alpha; MCD: methionine choline deficient; NASH: non‐alcoholic steatohepatitis; TRIM13: tripartite motif containing 13, a STING‐specific E3 ubiquitin ligase; uPAR: urokinase plasminogen activated receptor.

In summary, CAR‐M has shown promising therapeutic potential in liver non‐tumor diseases. Moving forward, to facilitate the clinical translation of this technology, future efforts should focus on optimizing the targeting and biosafety of delivery vectors, developing strategies for in vivo direct CAR‑M generation, and elucidating the long‑term residence and functional fate of CAR‑M in liver regeneration.

### 4.2 CAR‐M in Cardiovascular Diseases

Cardiovascular diseases (CVDs) represent one of the leading causes of death worldwide and are expected to impose an increasing global health burden in the coming decades [150]. Their development is associated with multiple risk factors, including physical inactivity, unhealthy nutrition, and dyslipidemia [151]. Although different CVDs present distinct clinical manifestations, they often share some common pathological hallmarks, such as cardiac fibrosis and chronic inflammation [152, 153]. Current therapeutic strategies for CVDs mainly involve medicines and surgical treatments, which have improved patient outcomes and reduced mortality to some extent [154]. However, these approaches remain limited by their insufficient capacity for cardiac tissue regeneration, continued reliance on sophisticated palliative therapies, and potential adverse effects. To address these problems, the development of more effective tissue engineering strategies remains an important goal in cardiovascular research [155].

**FIGURE 8:**
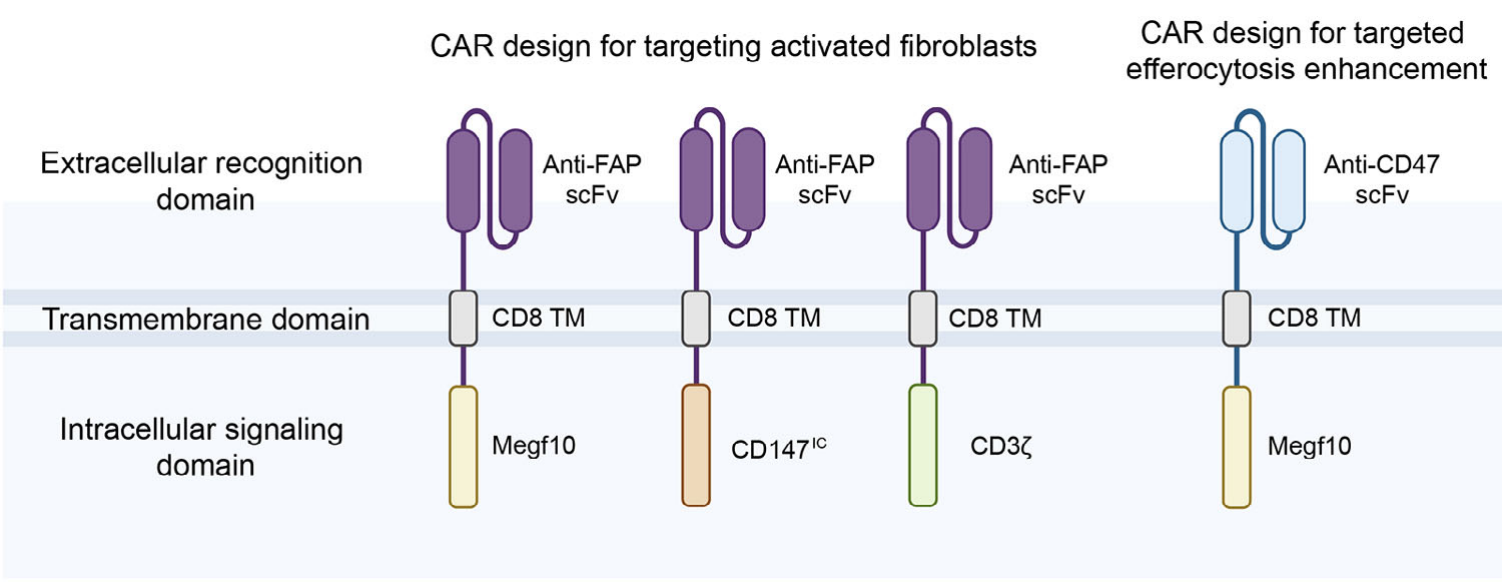
Validated CAR designs for macrophage engineering in CVDs. Anti‐FAP scFv directs engineered macrophages toward activated CFs to limit excessive ECM deposition and attenuate fibrosis progression. Anti‐CD47 scFv targets the “don't eat me” signal, CD47, which is upregulated on apoptotic cells in atherosclerotic lesions. The intracellular domains define distinct macrophage effector programs. Megf10 promotes antigen‐triggered phagocytosis, CD147^IC^ may enhance MMP‐associated collagen degradation, whereas CD3ζ is adapted from conventional CAR‐T designs to support macrophage activation and phagocytic responses. Created in BioRender. Cui, S. (2026) https://BioRender.com/co780cc.

The abilities of CAR‐M to mediate antigen‐directed phagocytosis and reprogram maladaptive inflammation make them a congruent therapeutic agent for CVDs. By coupling target‐specific recognition with tailored intracellular signaling, CAR constructs can program macrophages toward defined therapeutic functions. In the context of CVDs, current preclinical studies and proposed strategies mainly converge on two directions: anti‐FAP CARs for targeting activated cardiac fibroblasts (CFs) and anti‐CD47 CAR for enhancing efferocytosis (Figure 8).

#### 4.2.1 CAR‐M Alleviating Cardiac Fibrosis

Cardiac fibrosis is a common driver of pathological heart remodeling across various CVDs because fibroblast activation is a shared response to different forms of cardiac injury, including hypertensive cardiac damage, ischemia‐reperfusion (I/R) injury, and myocardial infarction (MI). Without timely intervention, fibrosis may increase ventricular stiffness, promote arrhythmias, and impair microvascular oxygen delivery, collectively accelerating heart failure progression [156]. Fibrosis development is governed by a sophisticated regulatory network involving profibrotic signaling, matrix‐integrin interactions, and newly discovered immune‐fibroblast crosstalk [157].

Although targeting upstream signaling cascades remains of substantial interest [158], their downstream effects ultimately converge on cardiac fibroblasts, the key executors of ECM deposition [159]. This convergence underscores the rationale for therapeutic strategies designed to eliminate myofibroblasts. Accordingly, CAR‐M‐based cell immunotherapy has emerged as an actively explored approach, leveraging the ability of engineered macrophages to phagocytose activated myofibroblasts and secrete ECM‐degrading enzymes such as matrix metalloproteinases (MMPs) [160].

Table 4 summarizes representative CAR‑M applications in distinct cardiac fibrosis models. Across these studies, two distinct engineering strategies have been pursued. Gao et al. employed lentiviral transduction to engineer mouse BMDMs expressing a FAP‐targeting CAR with a Megf10 intracellular domain to enhance phagocytic activity [161]. In a mouse model of angiotensin II and phenylephrine (AngII/PE)‐induced hypertensive cardiac injury, adoptive transfer of these CAR‐M significantly reduced FAP^+^ myofibroblasts and fibrotic area while improving cardiac function, without inducing systemic inflammation or long‐term adverse effects. Similarly, Wang et al. used lentiviral vector‐constructed CAR‐M to alleviate myocardial fibrosis in mice after I/R [162].

**TABLE 4:** Representative CAR‐M applications in cardiac fibrosis.

Disease model	Target cell or antigen	Macrophage type	Administration route	Delivery method	Therapeutic readout	Unresolved safety issue
Murine cardiac fibrosis model induced by continuous AngII/PE treatments (representing hypertensive cardiac injury) [161]	FAP^+^ myofibroblasts	Mouse BMDMs	Intravenous injection (5×10^6^ cells/mouse at 1‐ and 2‐week time points after first AngII/PE treatment)	Lentiviral transduction ex vivo	Reduced disease‐associated FAP^+^ cells; Reduced cardiac fibrosis; Improved cardiac function	Potential off‐target toxicity in non‐cardiac organs
Murine myocardial I/R injury induced by left anterior descending (LAD) coronary artery ligation and reperfusion [162]	FAP^+^ activated CFs	Mouse BMDMs	Intravenous injection (4×10^6^ cells/mouse on day 3 after I/R)	Lentiviral transduction ex vivo	Improved cardiac function after I/R; Reduced myocardial fibrosis and cleared activated fibroblasts; Maintains long‐term efficacy for at least 3 months	Potential long‐term arrhythmia and systemic organ toxicity; Immunogenicity due to long‐term persistence of CAR‐M
Murine MI model induced by LAD coronary artery ligation [163]	FAP^+^ activated CFs	Endogenous cardiac macrophages in the infarct region	Intravenous injection	LNP‐mediated in vivo dual mRNA delivery (CAR and Legumain)	Improved post‐MI survival rate and cardiac function; Suppressed ventricular dilation and hypertrophy; Reduced infarct size and myocardial fibrosis	Long‐term treatment safety; potential immunogenicity of repeated dosing
Murine myocardial I/R injury induced by LAD coronary artery ligation and reperfusion [41]	FAP^+^ activated CFs	Endogenous circulating monocyte‐derived macrophages	Intravenous injection (LNP dosage: 2 mg/kg at day 1 and day 3 post‐I/R)	LNP‐mediated in vivo delivery of FAP CAR mRNA	Reduced FAP^+^ activated fibroblasts and alleviated cardiac fibrosis; Decreased collagen III level; Improved cardiac function with both acute efficacy and sustained long‐term benefits	Potential systemic toxicity and off‐target organ accumulation after systemic LNP delivery

**FIGURE 9:**
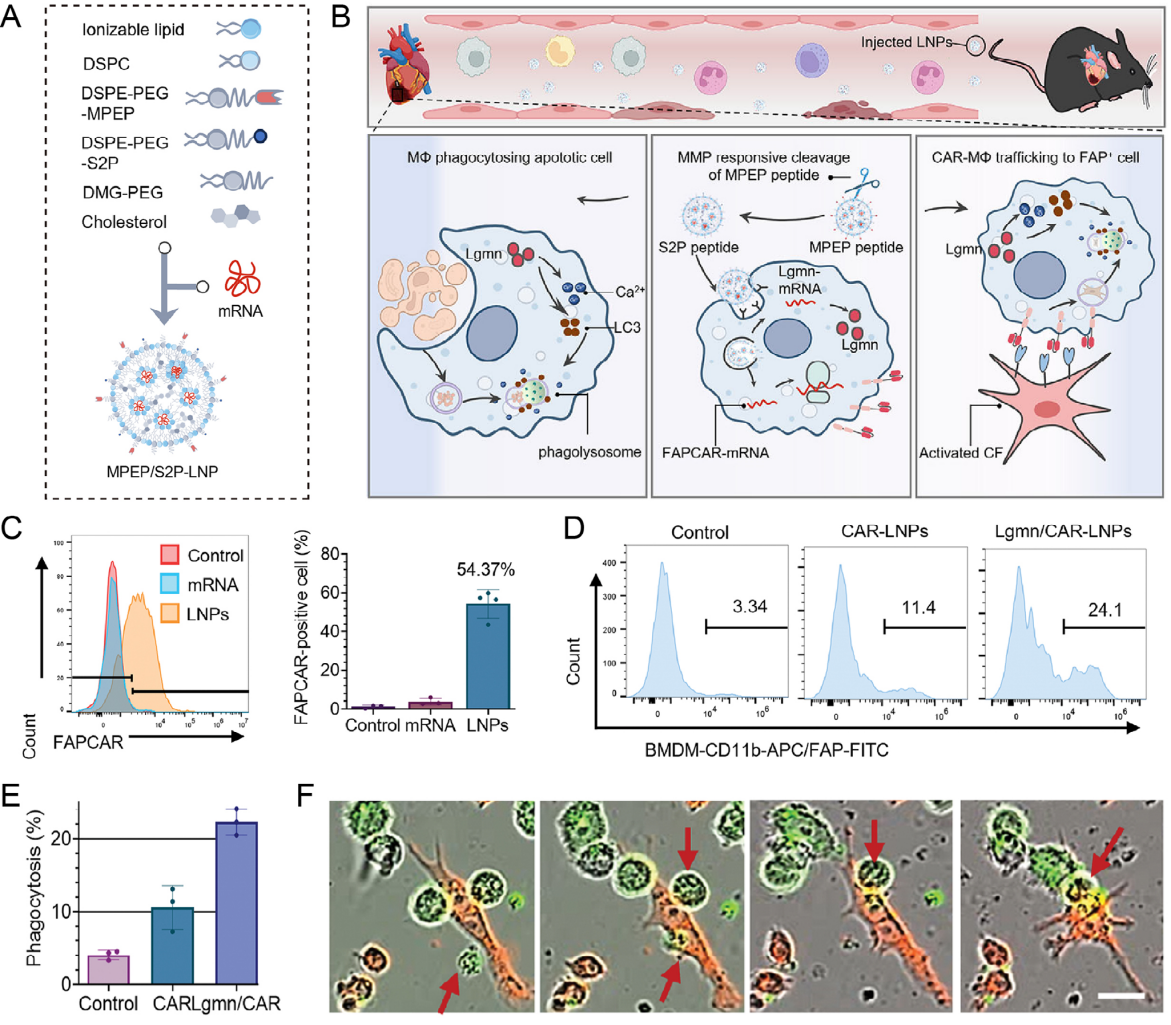
In vivo generation of CAR‐M via LNP platform reduces cardiac fibrosis after MI. (A) Schematic illustration of LNP preparation. (B) Schematic illustration of CAR‐M generation for the clearance of activated CFs. (C) Flow cytometric analysis of anti‐FAP expression in BMDMs 24 h after treatment with free mRNA or CAR‐LNPs. Quantitative analysis is shown on the right. (D) Representative flow cytometry plots showing phagocytosis of FAP^+^ target cells by macrophages treated with free mRNA, CAR‐LNPs, or Lgmn/CAR‐LNPs. (E) Quantitative assessment of phagocytic capacity. (F) Phagocytic activity of CAR‐M (red) cocultured with FAP^+^ target cells (green). Scale bars = 20 µm. Reproduced with permission [163]. Copyright 2025, Wiley‐VCH.

More recently, LNP‐mediated in vivo delivery has emerged as an alternative to circumvent the procedural complexity and high manufacturing costs of ex vivo cell engineering [163, 164]. Liu et al. developed a dual mRNA‐loaded LNP platform that directly generates fibrosis‐targeted CAR‐M within the pathological microenvironment [163]. Through a surface engineering strategy, elevated MMP‐2/9 activity in the MI region selectively cleaves the infarct area‐targeted peptide (MPEP) on the LNPs, exposing S2P peptides that promote macrophage‐specific uptake via receptor‐mediated endocytosis (Figure 9A,B). This design achieved a 2.4‐fold increase in cardiac accumulation compared with non‐targeted LNPs. The LNPs co‐deliver FAP CAR mRNA and legumain (Lgmn, encoding an endolysosomal cysteine protease that enhances phagolysosomal cargo degradation) mRNA, thereby achieving both fibrosis targeting and macrophage efferocytosis (Figure 9C–E). Following in vitro confirmation of CAR‐M‐mediated engulfment of FAP^+^ target cells (Figure 9F), animal studies demonstrated that Lgmn/CAR treatment improved 4‐week post‐MI survival rate from 45.00% to 81.25%.

Du et al. developed macrophage‐targeted LNPs encapsulating FAP CAR mRNA, building upon the ALC‐0315 platform with the incorporation of 1,2‐dioleoyl‐sn‐glycero‐3‐phospho‐L‐serine (DOPS) [41]. DOPS can be recognized by scavenger receptors highly expressed on macrophages to promote preferential uptake. In a murine myocardial I/R model, this treatment significantly decreased FAP‐positive fibroblasts and improved cardiac function compared with controls, with benefits sustained for at least two months post‐injury.

Collectively, these studies validate FAP as a target for activated CFs across multiple injury contexts. While adoptive transfer of lentivirally engineered CAR‐M has shown substantial efficacy, LNP‐mediated in vivo delivery offers a promising path toward more clinically feasible strategies. Although these methods have shown preliminary safety, their long‐term off‐target effects and potential immunogenicity of repeated dosing warrant further investigation.

#### 4.2.2 CAR‐M Therapy for Atherosclerosis

Atherosclerosis is another prevalent form of CVDs, characterized by lipid deposition and chronic inflammation within large arteries [165]. During disease progression, macrophages internalize excessive oxidized low‐density lipoprotein and transform into foam cells, which lead to plaque formation [165]. These foam cells undergo apoptosis as lesions develop; however, their timely clearance is impaired by defective efferocytosis [166], driven by upregulation of the “don't eat me” signal CD47 on apoptotic cells (ACs) and proteolytic cleavage of efferocytotic receptors such as MerTK on macrophages [167, 168]. Accumulation of uncleared ACs drives necrotic core expansion, which is a hallmark of advanced plaques [169]. Concurrently, heightened oxidative stress amplifies inflammation and lipid oxidation, which further reinforces plaque progression and instability [170].

**FIGURE 10:**
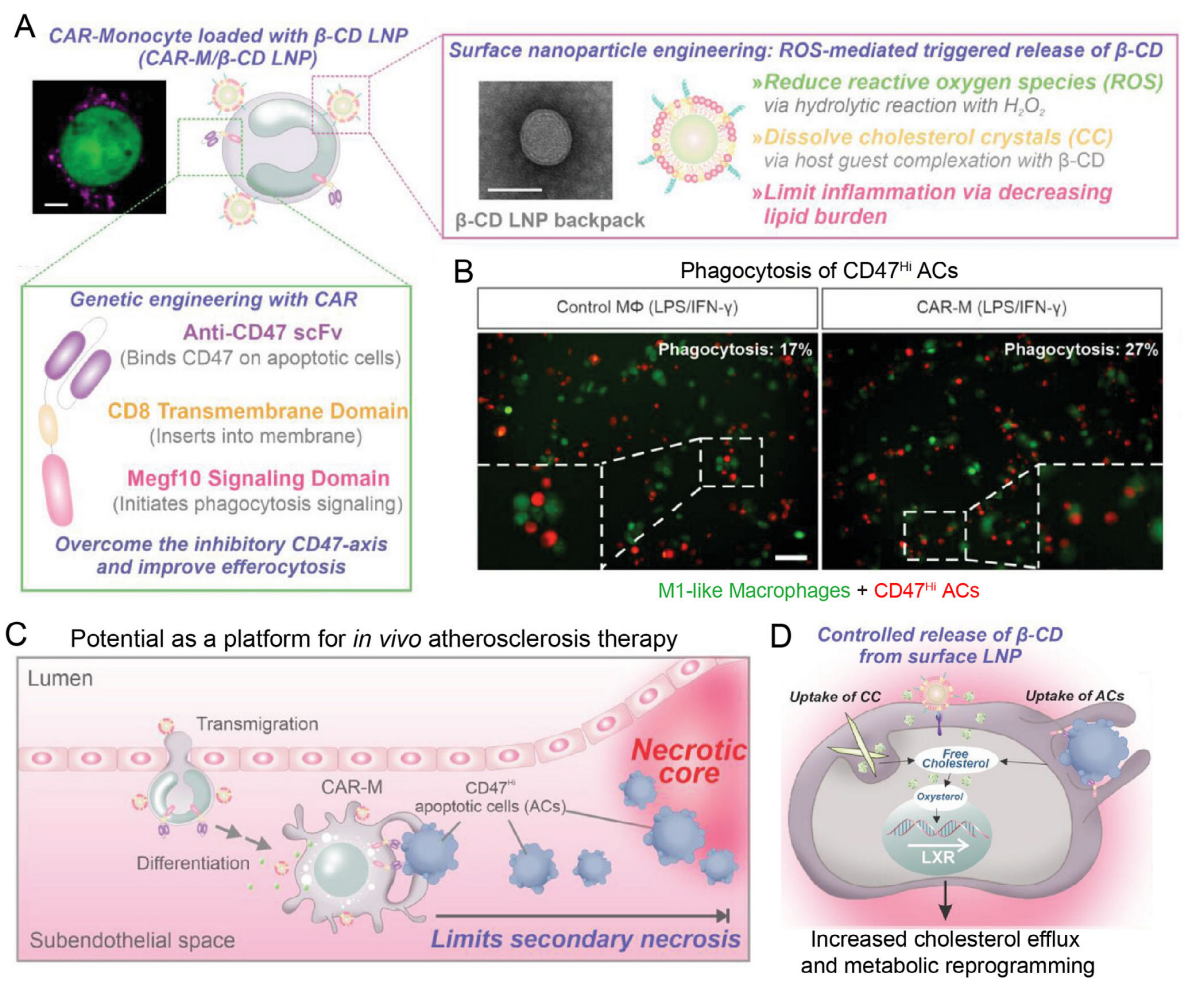
LNP‐anchored CAR‐M with antigen‐specific efferocytosis promotes atherosclerotic plaque clearance. (A) CAR‐transduced THP‐1 monocytes conjugated with β‐CD LNPs with the design layout of the anti‐CD47 CAR construct and β‐CD LNP. Left inset: confocal image of a CAR‑ monocyte carrying β‑CD LNP backpacks (scale bar = 5 µm). Right inset: TEM image of a β‑CD LNP (scale bar = 100 nm). (B) In vitro phagocytosis assay using M1 macrophages co‑cultured with CD47^Hi^ ACs. Scale bar = 100 µm. (C) β‐CD LNP‐anchored CAR‐THP1 cells home to inflamed lesions, differentiate, and selectively clear apoptotic cells. (D) Upon ROS‐mediated degradation of cell‐surface LNPs, the released β‐CD scavenges uptake ACs and cholesterol crystals. The resulting oxysterols subsequently activate the LXR signaling pathway. Reproduced under terms of the CC‐BY 4.0 license [171]. Copyright 2024, Wiley‐VCH.

Previous efforts to restore efferocytosis have relied on CD47‐blocking antibodies [172], but CAR‐M therapy advances this strategy by incorporating CD47 recognition into the CAR construct. For instance, Chuang et al. equipped CD47‐targeted CAR‐monocytes with surface‐anchored, ROS‐responsive β‐cyclodextrin (β‐CD) LNPs (Figure 10A) [171]. After 2 h of co‐culture with high CD47 expression ACs (CD47^Hi^ ACs), the engineered M1 CAR‐M achieved efficient AC clearance (approximately 27% compared to 17% in the control M1 macrophages) (Figure 10B). Once the CAR‐expressing monocytes home to the inflamed lesions, they differentiate into CAR‐M and rapidly clear ACs to limit secondary necrosis (Figure 10C). Simultaneously, the ROS‐sensitive LNP design allows spatiotemporally controlled release of β‐CD in the oxidative plaque microenvironment. Released β‐CD solubilizes cholesterol crystals and is internalized together with ACs into CAR‐M (Figure 10D). Intracellularly, β‐CD enhances cholesterol metabolism and activates the liver X receptor (LXR) signaling pathway in CAR‐M [173]. LXR activation promotes cholesterol removal from lesion foam cells, supports a tolerogenic macrophage response through efferocytosis, and dampens inflammation. Upon co‐culture with ACs, macrophages carrying β‐CD LNPs upregulated the LXR target genes *ABCA1* and *ABCG1* by 1.81‐ and 5.22‐fold, respectively, compared with unmodified macrophages. This was further accompanied by upregulation of genes associated with apoptotic cell clearance (*MERTK*, 1.57‐fold) and anti‐inflammation (*IL10*, 5.22‐fold). Collectively, this combined anti‐CD47 CAR‐M and β‐CD LNP platform synergistically restores efferocytosis while suppressing inflammation, outperforming CD47 blockade alone.

As shown in Table 5, this proof‑of‑concept study was conducted in a two‑channel vessel‑on‑a‑chip model of the atherosclerotic plaque subendothelial microenvironment rather than in an animal model. While the platform demonstrates promising in vitro efficacy, in vivo validation, including assessment of therapeutic efficacy, biodistribution, and long‑term safety, is urgently needed.

**TABLE 5:** Representative CAR‐M applications in atherosclerosis.

Disease model	Target cell or antigen	Macrophage type	Administration route	Delivery method	Therapeutic readout	Unresolved safety issue
A two‐channel vessel‐on‐a‐chip model of the atherosclerotic plaque subendothelial microenvironment [171]	Phagocytosis‐resistant CD47^Hi^ ACs	Human monocytic cell line THP‐1‐derived macrophages	Intravenous administration (proposed)	Lentiviral transduction ex vivo	Enhanced efferocytosis of CD47^Hi^ ACs by surface‐anchored ROS‐responsive β‐CD LNP backpacks; Improved LXR‐mediated cholesterol efflux Reduced cholesterol‐crystal and intracellular lipid accumulation, limiting foam‐cell formation	Not evaluated (in vitro model; in vivo safety remains to be determined)

In summary, these findings demonstrate that CAR‐M therapy can effectively counteract pathological cardiovascular remodeling. Notably, the success of these approaches relies heavily on the delivery strategy. While ex vivo lentiviral engineering provides robust and stable CAR expression, the emerging LNP‐based in vivo platforms offer a more clinically appealing route by bypassing ex vivo manipulation and enabling localized mRNA delivery. Importantly, the optimization strategies discussed before (such as surface targeting and microenvironment‐responsive release) are directly exemplified in these cardiovascular applications. Building on this foundation, future integration with biomaterial scaffolds or other delivery platforms may further enhance CAR‐M persistence and functional longevity within injured tissues.

## 5 Difference Among CAR‐M, CAR‐T, and CAR‐NK

CAR‐M has demonstrated significant potential in promoting tissue repair, particularly in conditions characterized by fibrosis, impaired efferocytosis, or inflammation. Unlike pharmacological interventions that primarily inhibit pathological progression or provide temporary tissue protection, CAR‐M therapy actively remodels the pathological microenvironment toward curative outcomes.

Given the rapid development of other cell therapy platforms, most notably CAR‐T and CAR‐NK therapies, it is essential to position CAR‐M within the broader therapeutic landscape. CAR‐M offers several unique strengths over these alternatives. First, macrophages exhibit superior infiltration into dense tissues, such as fibrotic tissue or atherosclerotic plaques [161, 163]. Second, CAR‐M therapy reduces the risk of cytokine release syndrome (CRS) [174]. Third, CAR‐M can exploit macrophage‐intrinsic phagocytic machinery to clear abnormal cells or structures [171]. Table 6 summarizes the mechanisms, advantages, and disadvantages of CAR‐M, CAR‐T, and CAR‐NK. Despite these strengths, CAR‐M therapy faces two major barriers. First, macrophages exhibit limited proliferative capacity and transient persistence in vivo, often requiring repeated dosing to achieve sustained therapeutic effects [34, 175]. Second, the intrinsic phenotype plasticity of macrophages discussed in Section 2 raises concerns about functional stability after infusion or in vivo generation. Unlike CAR‐T cells, CAR‐M can be rapidly reprogrammed by the pathological microenvironment, potentially eroding their engineered functionality. These interconnected limitations represent central hurdles for the field.

**TABLE 6:** A comparative summary of CAR‐M, CAR‐T, and CAR‐NK therapeutic strategies.

c	Mechanism	Advantages	Disadvantages
CAR‐M	CAR‐dependent phagocytosis	● Enhanced tissue infiltration ● Reduced CRS risk	● Less potent immediate cytotoxicity ● Phenotype plasticity due to microenvironmental cues ● Limited proliferative capacity
CAR‐T	CAR‐dependent cytotoxic killing via perforin/granzyme release	● Potent target cell killing ● Long‐term persistence	● Poor tissue penetration ● High CRS risk ● High manufacturing cost
CAR‐NK	Dual killing mechanism: CAR‐dependent and independent cytotoxicity	● Off‐the‐shelf availability from allogeneic sources	● Potential dependency on continuous cytokine administration for cell proliferation

## 6 Future Engineering Directions of CAR‐M Therapies

Despite the promise of CAR‐M therapies for tissue repair, several critical bottlenecks continue to impede their efficacy and clinical translation. Currently, the field is constrained by inefficient cell delivery and manufacturing, limited proliferation and transient persistence, insufficient macrophage‐specific design, and phenotypic instability within pathological microenvironments. Overcoming these multi‐dimensional limitations is pivotal for transitioning CAR‐M platforms from bench concepts to robust, clinically viable therapeutics. To this end, this section discusses the pivotal engineering directions designed to address these bottlenecks, focusing on delivery strategies, macrophage‐specific signaling optimization, metabolic modulation, and the establishment of a feasible roadmap for future development.

### 6.1 Delivery Strategy: Controlling Where and How CAR‐M are Generated

The choice between ex vivo engineering and in vivo programming has direct implications for the safety, controllability, persistence, manufacturing feasibility, and clinical applicability of CAR‐M therapy (Table 7). In ex vivo engineering, isolated macrophages or macrophage precursors are genetically modified outside the body and then administered as functional cellular products [176]. This strategy offers a high degree of controllability, as cell dose, CAR expression level, and macrophage phenotype can be characterized before infusion. It is also less dependent on the abundance and functional status of endogenous macrophages in diseased tissues [177]. However, ex vivo engineered CAR‐M may broadly distribute to non‐target organs after systemic administration due to the intrinsic trafficking properties of macrophages, raising off‐target safety concerns [149, 161]. The limited proliferative capacity of macrophages, donor‐to‐donor heterogeneity, and complex cell processing procedures further constrain scalable manufacturing [34, 176, 178].

**TABLE 7:** Comparison of ex vivo engineering and in vivo programming strategies for CAR‐M therapies.

	Ex vivo engineering	In vivo programming
Basic principle	Macrophages are isolated, engineered, characterized, and reinfused.	CAR‐encoding payloads are delivered directly into the body to program endogenous macrophages in situ.
Safety profiles	Possible off‐target tissue accumulation after infusion; integrating vectors pose insertional mutagenesis risks.	Avoids live‐cell infusion; can use non‐integrating mRNA. Off‐target delivery remains a concern.
Controllability	Cell dose, CAR expression, and initial phenotype can be predefined.	Vehicle dose is controllable, but the number, location, and persistence of generated CAR‐M are less predictable.
Manufacturing complexity	High complexity and cost; requires cell isolation, engineering, expansion, and quality control.	Low complexity; avoids live‐cell processing and supports scalable off‐the‐shelf production.
Clinical applicability	Suitable when precise product control is required, when endogenous macrophages are depleted, or when durable CAR expression is desired.	Suitable for acute/subacute conditions, repeated transient intervention, or diseases with sufficient endogenous macrophage pools.

To address these limitations, increasing attention has turned to in vivo programming using advanced delivery platforms. By tailoring carrier composition, surface ligands, and tissue‐targeting properties, these systems can deliver CAR‐encoding mRNA directly to macrophage populations within pathological niches [179]. This strategy avoids complex live‐cell processing and supports off‐the‐shelf production. Non‐integrating mRNA delivery enables transient CAR expression, which may be advantageous in tissue repair settings where prolonged macrophage activation could cause collateral damage.

Nevertheless, in vivo programming introduces a different set of challenges. Although the administered dose of the delivery vehicle can be controlled, the number, anatomical distribution, phenotype, and persistence of CAR‐M generated in vivo remain difficult to predict. Systemic administration of nanocarriers frequently leads to accumulation in clearance organs such as the liver and spleen, which may limit site‐specific programming. In addition, this strategy depends on the presence of sufficient and functionally competent endogenous macrophages, which may be depleted or impaired in chronic inflammatory or fibrotic diseases [81, 177]. These limitations highlight the need for multifunctional carriers that can improve macrophage selectivity, control expression duration, and modulate the local tissue environment to support stable CAR‐M generation and persistence [180].

The selection of an engineering route should be guided by the disease context and therapeutic objective. Ex vivo engineering is preferable when precise control over cell production is critical, when endogenous macrophages are insufficient, or when durable CAR expression is required. In addition, this strategy may be more appropriate for chronic or refractory diseases in which individualized cell manufacturing is justified. However, its high cost and complex logistics limit broad application in routine tissue‐repair settings. In vivo programming is better suited for acute conditions, transient modulation, or indications where repeated dosing is acceptable. Its scalability and off‐the‐shelf nature make it attractive for broader clinical use. However, this strategy is less appropriate when endogenous macrophage populations are severely depleted or located in tissues that are difficult to access by delivery vehicles. Importantly, these two strategies are not mutually exclusive. Hybrid strategies combining ex vivo engineering with biomaterial scaffolds or local delivery matrices may help improve tissue retention and prolong CAR‐M persistence.

### 6.2 CAR Design and Signaling Optimization: Improving Macrophage Specificity and Reducing Off‐Target Effects

While delivery strategy determines where and how CAR‐M are generated, CAR design directly determines how macrophages respond and their phenotype after antigen recognition. A major current bottleneck is that many CAR constructs used in macrophages are largely adapted from CAR‐T designs. Although these constructs have shown efficacy in certain settings, they may not optimally engage macrophage‐specific effector programs [33]. Unlike T cells, macrophages exert therapeutic functions through phagocytosis, efferocytosis, cytokine secretion, matrix remodeling, and phenotype transition [19]. Therefore, future CAR‐M design should prioritize intracellular signaling domains that more precisely harness macrophage‐intrinsic functions rather than simply maximizing immune activation [33].

In this regard, developing macrophage‐specific signaling modules represents a pivotal direction for functional optimization. Recent antitumor studies have successfully employed dual intracellular signaling domains to synergistically combine target engulfment with M1 polarization [33]. Building on this design principle, a promising strategy for tissue repair would be to pair clearance capacity with pro‐reparative M2 polarization signals. However, whether such dual‐domain constructs can effectively coordinate therapeutic programs in tissue‐injury contexts has yet to be systematically evaluated.

Beyond intracellular signaling, the antigen‐recognition module also requires further refinement. Current CAR‐M strategies for non‐tumor diseases typically rely on single‐antigen recognition. However, many candidate targets in tissue repair, such as activated fibroblast markers, ECM components, or inflammatory mediators, may also be transiently expressed during physiological wound healing or in non‐target tissues [181]. This creates a risk of off‐target recognition and excessive tissue remodeling. To improve specificity and safety, emerging multi‐CAR strategies incorporating Boolean‐logic gating may enable macrophages to integrate multiple disease‐associated signals and restrict full activation to cells or tissues that co‐express defined combinations of markers [182]. In addition, inducible CAR systems, tunable activation thresholds, or safety‐switch designs may further improve controllability and reduce unintended activation in healthy reparative tissues.

### 6.3 Metabolic Modulation: Stabilizing the Engineered Phenotype and Steering Polarization

If CAR design defines what macrophages are programmed to do, metabolic modulation determines how long they can sustain this function and whether they can maintain the desired phenotype under pathological stress. As discussed in Section 2, injured tissues are often characterized by hypoxia, lactate accumulation, acidic pH, oxidative stress, and lipid overload [44, 45, 46, 47]. These metabolic pressures can impair mitochondrial fitness, disturb redox homeostasis, alter macrophage polarization, and weaken phagocytic or efferocytic capacity [52, 53]. Therefore, even when CAR‐M are successfully generated or delivered to injured tissues, their engineered functions may be compromised by the local metabolic microenvironment.

Future CAR‐M engineering should therefore incorporate metabolic adaptability as an important design parameter. Potential strategies include metabolic preconditioning before adoptive transfer, co‐delivery of pathway‐tuning agents that support mitochondrial fitness, selection of intracellular signaling domains that favor reparative metabolic states, and integration with biomaterial‐based platforms that locally modulate inflammation and metabolic stress. For tissue‐repair applications, the ultimate goal of these metabolic interventions is not to broadly enhance macrophage activation, but to sustain the metabolic plasticity required to support stage‐appropriate transitions. In acute injury models, this involves supporting the dynamic transition from glycolysis‐driven clearance of necrotic debris during the early inflammatory phase to oxidative metabolism‐supported tissue resolution. Conversely, in chronic fibrotic environments, the priority shifts to restraining the aberrant glycolysis that fuels pro‐inflammatory macrophage polarization, while sustaining the metabolic pathways necessary for structure remodeling and scar resolution.

Although several metabolic modulation strategies have been explored in tumor immunotherapy and macrophage biology [98, 183], direct evidence for metabolic engineering of CAR‐M in tissue‐repair models remains limited. Future studies should therefore evaluate CAR‐M persistence, polarization, phagocytosis, efferocytosis, and tissue‐repair outcomes under disease‐mimicking metabolic conditions, including hypoxia, high lactate, lipid overload, oxidative stress, and inflammatory cytokine exposure. Such studies will be essential for determining whether metabolic modulation can preserve CAR‐M phenotypic fidelity and prolong durable reparative activity in injured tissues.

### 6.4 Perspective Developing Roadmap for Clinical Translation

**FIGURE 11:**
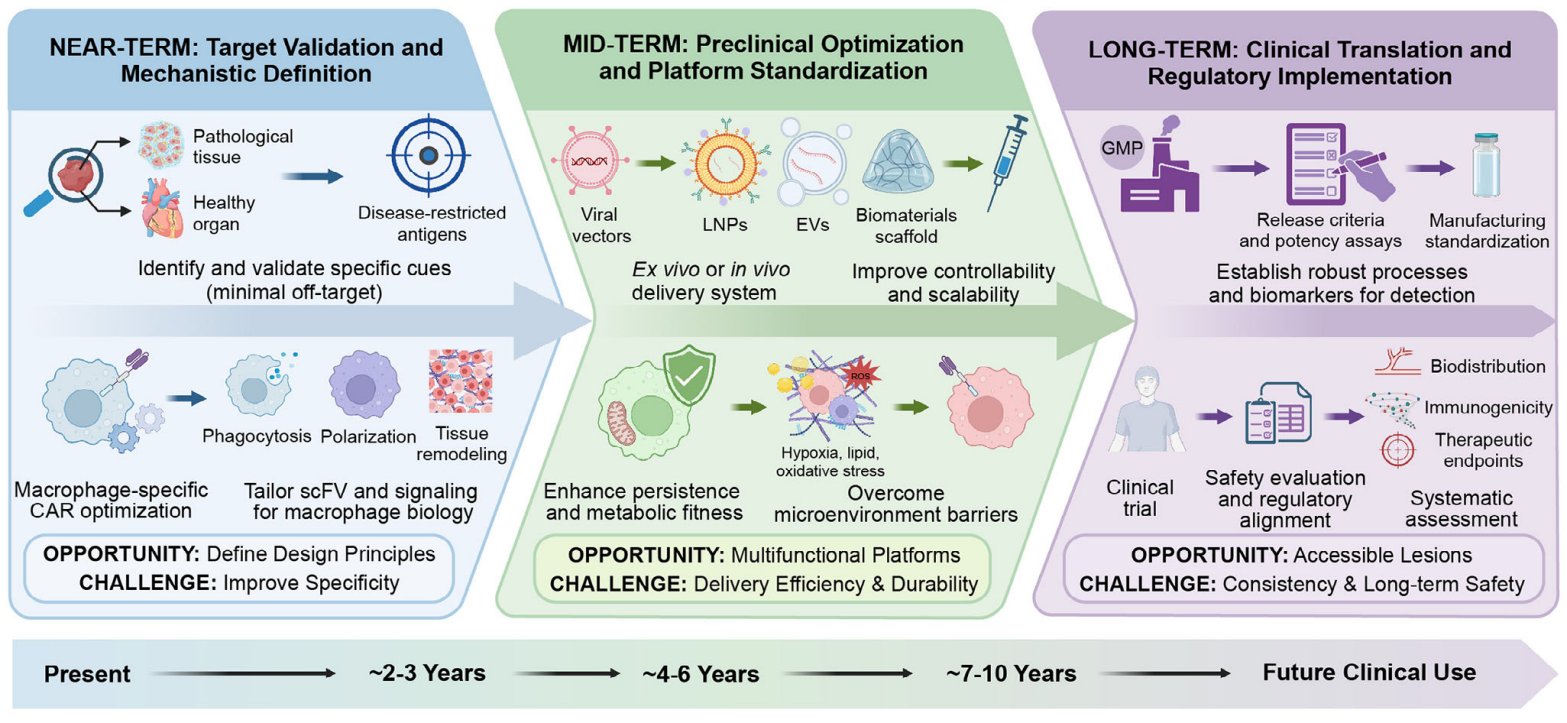
Roadmap for the development of CAR‐M therapies over the coming decade. Created in BioRender. Cui, S. (2026) https://BioRender.com/co780cc.

Over the next 5–10 years, CAR‐M therapy is expected to evolve from early proof‐of‐concept studies toward more standardized and clinically deployable platforms. As summarized in Figure 11, this developmental pathway can be divided into three overlapping stages: near‐term target validation and mechanistic definition, mid‐term preclinical optimization and platform standardization, and long‐term clinical translation and regulatory implementation.

In the near term, research should focus on consolidating the biological foundation of CAR‐M therapies for tissue repair. A central priority is to identify and validate disease‐restricted antigens or microenvironmental cues that are highly enriched in pathological tissues but minimally expressed in healthy organs. This is particularly important for tissue‐repair applications, where many candidate targets, such as activated fibroblast markers, extracellular matrix components, or inflammatory mediators, may also appear during physiological wound healing. In parallel, CAR architecture should be optimized specifically for macrophage biology rather than directly adapted from CAR‐T designs. Key questions include how different scFv, transmembrane, and intracellular signaling domains regulate macrophage activation, phagocytosis, efferocytosis, polarization, cytokine secretion, and tissue‐remodeling functions. At this stage, the major opportunity lies in defining macrophage‐specific design principles, whereas the main challenge is to improve target specificity and minimize off‐target recognition in normal reparative tissues.

In the mid‐term, the field will likely shift from target discovery and proof‐of‐concept validation toward preclinical optimization of engineering and delivery platforms. Although ex vivo viral transduction remains valuable for mechanistic studies and early therapeutic validation, future development may increasingly rely on non‐viral, transient, and in vivo delivery systems to improve controllability, repeat‐dosing feasibility, and manufacturing scalability. Advanced LNPs, EVs, polymeric nanocarriers, and biomaterial‐based local delivery systems may be further engineered to enhance tissue tropism, macrophage selectivity, endosomal escape, and microenvironment‐responsive release. At the same time, improving CAR‐M persistence, metabolic fitness, and phenotypic stability will be critical, especially in injured tissues. The key opportunity during this stage is to integrate delivery engineering, CAR signaling optimization, and metabolic modulation into multifunctional CAR‐M platforms. The major challenges include limited delivery efficiency, uncontrolled tissue distribution, variable macrophage states, and insufficient durability of reparative functions.

In the long term, successful clinical translation will require rigorous standardization, regulatory alignment, and systematic safety evaluation. Essential steps include establishing Good Manufacturing Practice (GMP)‐compatible workflows, defining release criteria, developing robust potency assays, and validating biomarkers that can predict CAR‐M biodistribution, persistence, activation state, and therapeutic efficacy. For in vivo programming strategies, particular attention should be paid to carrier biodistribution, off‐target transfection, repeat‐dose immunogenicity, innate immune activation, and long‐term tissue safety. For ex vivo engineered CAR‐M, critical issues include batch‐to‐batch consistency, donor variability, cell product characterization, persistence after infusion, and off‐target organ accumulation. Early clinical applications are most likely to emerge in indications with accessible lesions, well‐defined pathological targets, measurable therapeutic endpoints, and a strong unmet medical need.

Overall, the translational evolution of CAR‐M therapy for tissue repair should not merely aim to maximize activation, but to achieve stage‐specific, context‐appropriate, and controllable reparative functions. By integrating macrophage‐specific CAR signaling, disease‐adapted advanced delivery systems, and metabolic programs that preserve phenotypic fidelity, CAR‐M therapies can evolve from experimental tools into a new class of living medicines capable of driving durable tissue regeneration and resolving complex pathologies.

## 7 Conclusions

CAR‐M technology uniquely combines the innate phagocytic, tissue‑remodeling, and immunomodulatory functions of macrophages with CAR‑mediated precise targeting, offering distinct advantages in tissue engineering and regenerative medicine. This review systematically reviewed diverse delivery systems ranging from viral vectors to smart nanomaterials and biomaterial scaffolds. Among them, LNPs stand out due to advances in ionizable lipid chemistry, surface targeting modifications, and optimization of physicochemical properties, which have greatly improved the efficiency and safety of both ex vivo and in vivo CAR‐M programming. Importantly, the chemical properties of delivery systems not only affect transfection efficiency but also shape endocytic pathways, intracellular fate, and macrophage polarization status, thereby co‑determining the therapeutic performance of CAR‐M. In models of liver and cardiovascular diseases, CAR‐M have successfully demonstrated specific clearance of activated HSCs, myofibroblasts, and ACs, leading to reversal of fibrosis, improved cardiac function, and stabilization of atherosclerotic plaques. Compared with CAR‐T and CAR‐NK, CAR‐M exhibit superior tissue infiltration, lower risk of CRS, and pro‑repair functions. Nevertheless, phenotypic instability, insufficient in vivo persistence, and potential off‑target effects remain major barriers to clinical translation. Future research should focus on developing macrophage‑tailored CAR signaling domains, employing multi‑target logic gating for enhanced specificity, achieving organ‑specific or pathology‑responsive in situ programming, and improving metabolic adaptability to stabilize CAR‐M function. The deep integration of chemistry, materials science, and immune engineering will drive CAR‐M toward standardized therapeutic solutions, offering transformative regenerative immunotherapies for intractable tissue injuries including fibrosis, ischemic damage, and degenerative diseases.

## Author Contributions

**Yixin Zhang**: conceptualization, methodology, validation, writing – review and editing, writing – original draft, investigation, software. **Jintian Yu**: methodology, Writing – review and editing, Writing – original draft, validation, software. **Aikemu Ainiwaer**: supervision, resources. **Lingna Chen**: supervision, resources. **Liang Yuan**: resources, supervision. **Ziliang Fu**: writing – review and editing, validation. **Yiming Yao**: methodology. **Shixiong Chen**: supervision, resources. **Xiaolin Cui**: conceptualization, software, methodology, validation, writing – review and editing, supervision, resources.

## Conflicts of Interest

The authors declare no conflicts of interest.

## Data Availability

This article did not generate any new datasets. All data used are derived from previously published studies cited in this review.
